# Unleashing the potential of adipose organoids: A revolutionary approach to combat obesity-related metabolic diseases

**DOI:** 10.7150/thno.93919

**Published:** 2024-02-25

**Authors:** Xingran Liu, Jing Yang, Yuxin Yan, Qingfeng Li, Ru-Lin Huang

**Affiliations:** 1 Department of Plastic and Reconstructive Surgery, Shanghai Ninth People's Hospital, Shanghai Jiao Tong University School of Medicine, Shanghai, China.; 2 Shanghai Institute for Plastic and Reconstructive Surgery, Shanghai, China.

**Keywords:** Adipose organoid, Obesity, Type 2 diabetes mellitus, Metabolic disease, Brown adipose tissue

## Abstract

Obesity-related metabolic diseases, including obesity, diabetes, hyperlipidemia, and non-alcoholic fatty liver diseases pose a significant threat to health. However, comprehensive pathogenesis exploration and effective therapy development are impeded by the limited availability of human models. Notably, advances in organoid technology enable the generation of adipose organoids that recapitulate structures and functions of native human adipose tissues to investigate mechanisms and develop corresponding treatments for obesity-related metabolic diseases. Here, we review the general principles, sources, and three-dimensional techniques for engineering adipose organoids, along with strategies to promote maturation. We also outline the application of white adipose organoids, primarily for disease modeling and drug screening, and highlight the therapeutic potential of thermogenic beige and brown adipose organoids in promoting weight loss and glucose and lipid metabolic homeostasis. We also discuss the challenges and prospects in the establishment and bench-to-bedside of adipose organoids, as well as their potential applications.

## 1. Introduction

Obesity is a chronic metabolic disease characterized by excessive accumulation of adipose tissue which usually results in a low-grade, chronic, systematic inflammation state 1. Its worldwide prevalence has nearly tripled since 1975 2 and affects more than 1 billion people worldwide in 2022 3. Moreover, over 4 million people died each year as a result of being overweight or obese 4. According to the World Health Organization, obesity is defined as a body mass index over 30 kgm^2^, which is calculated by taking a person's weight, in kilograms, divided by their height, in meters squared 5. Obesity significantly increases the risk of dyslipidemia and insulin-resistance 1, which contribute to the development of type 2 diabetes mellitus (T2DM), non-alcoholic fatty liver disease (NAFLD), and cardiovascular diseases 6,7. These metabolic diseases are called obesity-related metabolic diseases (OMDs) 8,9. Mechanistically, high-fat and high-glucose diets cause energy intake to exceed energy expenditure, thereby contributing to OMD development 10,11. The pathogenesis of OMDs is not fully understood and current management includes lifestyle intervention challenging to keep 12, as well as medications and surgical interventions with undesired adverse effects 12,13. However, the lack of authentic and unlimited human models hampers further exploration of pathogenesis and the development of rational therapies with high efficacy and safety.

Organoids are three-dimensional (3D) tissue aggregates derived from stem cells, progenitor cells, and/or differentiated cells that self-organize through cell-cell and cell-extracellular matrix (ECM) interaction to recapitulate structures and functions of native tissue in vitro 7,14. They are miniaturized and simplified model systems of organs that have gained enormous interest for disease modeling, drug screening, and regenerative medicine 15-17. For OMDs, white adipose organoid (WAO) holds promise in disease modeling to decipher pathogenesis and drug screening for novel drug development. Besides, transplantation of brown adipose organoid (BrAO) and beige adipose organoid (BeAO) exhibits long-term therapeutic potential for OMDs by eliciting weight loss and improving glucose and lipid metabolism. Notably, lifestyle interventions are highly accessible and cost-free options for OMD treatment; however, they necessitate significant personal initiative and a high level of self-discipline. Pharmacologic treatments offer hope to a broader range of OMD patients but require tolerance for the medications' high costs and potential gastrointestinal and psychiatric side effects, as well as ongoing adherence to prevent weight regain 12,18. Surgery is also a boon for individuals lacking initiative, with bariatric surgery currently demonstrating favorable outcomes in obesity management; nevertheless, it is invasive and associated with complications such as cholelithiasis. Unfortunately, there are no surgical options available for OMDs like T2DM and NAFLD 12. Transplantation of BrAO and BeAO, like surgery, has the advantage of independence on personal initiative and long-term compliance compared to lifestyle interventions and pharmacological treatments. Moreover, it presents the benefits of being less invasive and universally effective for OMDs compared to surgery.

In this review, we summarize the general principles, sources, and 3D techniques to fabricate adipose organoids, as well as approaches to promote maturation. We also review their current applications in obesity, T2DM, dyslipidemia, and NAFLD and discuss the challenges and prospects for further clinical applications.

## 2. Adipose organoid, an emerging tool to combat OMDs

To better establish adipose organoids, it is imperative to understand native adipose tissues first. Adipose tissue is a heterogeneous organ with a complex microenvironment and plays a pivotal role in energy hemostasis regulation. White adipose tissues (WATs) containing lipid-accumulated white adipocytes contribute to energy storage; whereas brown adipose tissues (BATs) and beige adipose tissues both contain adipocytes with high uncoupling protein 1 (UCP1) expression for energy-burning. Adipose tissues also contain mesenchymal stem cells (MSCs), endothelial cells (ECs), immune cells, and neurons 7,19. The ECM containing collagens and polysaccharides significantly impacts cell survival, adhesion, migration, differentiation, and metabolism 20. Moreover, adipose tissues secret adipokines, such as leptin, adiponectin, and batokines (adipokines from brown and beige fat) 21, which mediate communication with livers, skeletal muscles, and satiety centers. Therefore, adipose tissue is considered an endocrine organ, and several reviews have elaborated on adipokines 19,21-23.

For WAT characterized by unilocular adipocytes 7, it stores and releases energy in the form of fatty acids in response to systematic demands 24. Therefore, WAOs should contain adipocytes with a large unilocular lipid droplet 7, which can be characterized by scanning electron microscopy 25, H&E staining 25,26, AdipoRed 28, BODIPY 25,29, and Oil Red O staining 30, as well as perilipin immunostaining 31. WAOs should also express common adipogenic markers such as ADIPOQ, FABP4, PPARG, and C/EBPB, as well as specific white markers like LEP, RETN, and AGT 24. In terms of function, WAOs should at least possess the endocrine function in adipokine secretion 27,28. Besides, triglyceride accumulation and glycerol release corresponding to energy storage and release in vivo are also evaluated sometimes 28. Notably, excessive lipid accumulation in adipose tissues usually results in insulin-resistance, increased lipolysis, and pro-inflammatory cytokine secretion 23,32. The hypertrophic WATs contribute to the elevation of peripheral glucose and lipid levels, lipotoxicity in various organs, and further to OMD development 23,32. Therefore, through fatty acid exposure, TNF-α stimuli, or macrophage co-culture, WAOs can be successfully applied to disease modeling and drug screening of obesity, T2DM, and NAFLD.

Brown and beige adipocytes contain multiple small lipid droplets and numerous cristae-dense mitochondria with high UCP1 expression 21. Developing embryonically, BATs located in specific regions such as the interscapular region are present only in infancy in humans but are maintained in adulthood in mice 24,33. Beige adipocytes emerge sporadically in WATs postnatally in response to cold exposure or other certain stimuli 24. The multilocular structure of adipocytes in BrAOs and BeAOs is usually characterized by phase contrast imaging 34,35, scanning and transmission electron microscopy imaging 35, H&E staining 36, Oil Red O 37, LipidTox Green 35, and BODIPY staining 38, and perilipin immunostaining 39. Moreover, dense mitochondria with high UCP1 expression are usually characterized by immunostaining 35,36. They also express thermogenic markers such as UCP1, DIO2, CIDEA, PPARGC1A, and PRDM16 24. However, these are insufficient for distinguishing between brown and beige identities; hence, a test on the expression of specific markers is imperative. It was reported that LHX8, ZIC1, EVA1, and PDK4 are exclusive for classical brown adipocytes, while TBX1, CITED, SHOX2, CD137, and TMEM26 are for beige ones 24,35,40.

In terms of function, brown and beige adipose tissues convert energy into heat by burning fatty acids and glucose through uncoupling respiration. Besides, the batokines usually contribute to metabolic hemostasis through inter-organ communication 19. Therefore, BrAOs and BeAOs show high-level and β-adrenergic responsive glucose and lipid uptake, lipolysis, and glycolysis, and basal, proton-leak, and maximal oxygen consumption rates (OCR) in vitro 34-36. The glucose uptake and lipid uptake are assessed by measuring the radioactivity of [^3^H]-2-deoxyglucose 41 and [^14^C] palmitic acid 41, respectively, and lipolysis is quantified by glycerol release. Besides, the Seahorse bioanalyzer is often used to assess energy extracellular acidification rates for anaerobic glycolysis and OCR for aerobic mitochondrial respiration 35. BrAOs and BeAOs also secret batokines which improve insulin-resistance 39 and endogenous adipokine secretion 36. Therefore, the functions of non-shivering thermogenesis and metabolic healthy secretion enable BrAOs and BeAOs to treat OMDs after transplantation 7,21.

In practice, the term 'adipose organoid' has often been used interchangeably with other terms, such as 'adipose spheroid' 42, 'adipocyte aggregate' 43, 'fat organoid' 44, 'adipose tissue' 38,45, and 'adipose microtissue' 36. Considering the structures and functions they exhibited, we discuss them uniformly in the term 'adipose organoid' in the following section.

## 3. Strategies to establish adipose organoids

### 3.1 General principles to establish adipose organoids

**Figure 1:**
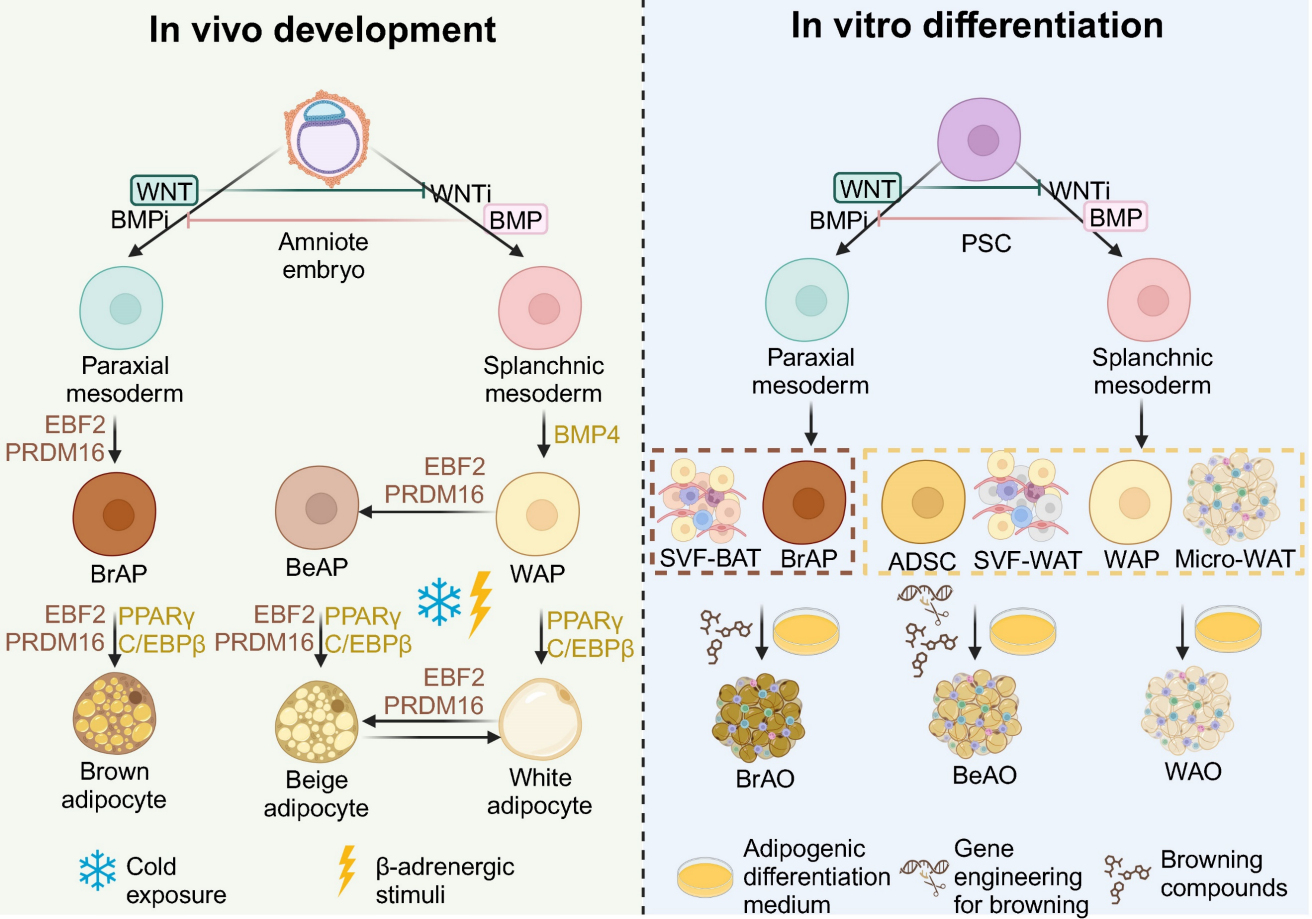
** General principles to generate adipose organoids in vitro based on the in vivo development of adipose tissues.** Based on the understanding of the development in vivo, by modulating WNT and BMP signaling pathways, brown and white adipocyte progenitors going through paraxial and splanchnic mesoderm, respectively, are induced from pluripotency stem cells (PSCs). Brown adipocyte progenitors from PSCs and stromal vascular fragments from brown adipose tissues correspond to the brown adipocyte progenitor stage in vivo which are fate-determined to brown adipocytes. Therefore, brown adipose organoids can be derived in the basic adipogenic differentiation medium through activation of PPARγ and C/EBPα, key factors of adipogenesis; while supplementation of browning compounds that mimic the EBF2 and PRDM16 activation in vivo can further enhance the differentiation efficiency. White adipocyte progenitors from PSCs, adipose-derived stem cells, stromal vascular fragments from white adipose tissues, and micro white adipose tissues correspond to the white adipocyte progenitor stage in vivo which is bipotent. Therefore, white adipose organoids can be derived in the basic adipogenic differentiation medium. With additional genetic engineering techniques to introduce thermogenic genes and/or supplementation of browning compounds to mimic the cold exposure or β-adrenergic stimuli that activate EBF2 and PRDM16 in vivo, beige adipose organoids can be successfully induced from these bipotent sources. ADSC: adipose-derived stem cell; BeAO: beige adipose organoid; BeAP: beige adipocyte progenitors; BrAO: brown adipose organoid; BrAP: brown adipocyte progenitor; Micro-WAT: micro white adipose tissue; PSC: pluripotent stem cell; SVF-BAT: stromal vascular fragment from brown adipose tissue; SVF-WAT: stromal vascular fragment from white adipose tissue; WAO: white adipose organoid; WAP: white adipocyte progenitor.

All three adipose tissues rely on the involvement of peroxisome proliferator-activated receptor γ (PPARγ) and CCAAT/enhancer binding protein α (C/EBPα) for adipogenesis, with their fate bifurcation occurring at an earlier stage. The development of the three adipose tissues is demonstrated in detail in **Figure 1**. In brief, at the embryonic stage, BATs derive from the dermomyotome of FOXC1^+^ MYF5^+^ PAX3/7^+^ paraxial mesoderm driven by EBF2 and PRDM16 24, whereas WATs originate from ISL1^+^ FOXF1^+^ splanchnic mesoderm (SplM) driven by BMP4 23. This specification of mediolateral fates of mesoderm is controlled by antagonism of the BMP and WNT signaling pathways 46,47. Beige adipose tissues are induced from WATs under cold exposure or β-adrenergic stimuli at the postnatal stage 24. The highly inducible brown-like adipocytes will go whitening to dormant white adipocytes if the stimuli are withdrawn while reinstalling into beige adipocytes after re-exposure to stimuli 21,24,33.

Based on the understanding of the development in vivo, various sources have been applied. State-of-the-art sources include PSCs corresponding to the amniote embryo stage in vivo, ADSCs, stromal vascular fragments (SVFs) from WAT (SVF-WATs), and micro-WATs corresponding to the white adipocyte progenitor stage, as well as SVF-BATs corresponding to the brown adipocyte progenitors stage. Therefore, PSC-derived white adipocyte progenitors, ADSCs, SVF-WATs, and micro-WATs exhibit bipotency. WAOs are derived in the common adipogenic differentiation medium with small molecules that activate PPARγ and C/EBPα; whereas BeAOs are generated with additional compounds or gene engineering techniques to mimic the external browning stimuli. PSC-derived brown adipocyte progenitors and SVF-BATs are unipotent to brown adipocytes after adipogenic differentiation. The supplementation of browning compounds could further enhance brown differentiation efficiency.

All sources undergo the adipogenic differentiation process with PPARγ and C/EBPα being activated synergetically 23,48. The adipogenic differentiation medium basically consists of insulin, dexamethasone, and 3-Isobutyl-1-methylxanthine (IBMX) with other adipogenic compounds supplemented sometimes. In detail, insulin activates AKT/mTOR signaling pathway 23,49, triiodothyronine (T3) activates thyroid hormone receptor 50, and indomethacin inhibits cyclooxygenase 51 to promote PPARγ activation. Meanwhile, rosiglitazone directly activates PPARγ 52. In addition, glucocorticoids such as dexamethasone activate C/EBPδ 23,36,53, cyclooxygenase inhibitors such as indomethacin activate CEB/Pβ 51, phosphodiesterase inhibitors such as IBMX increase cAMP 54,55, and TGF-β inhibitors such as SB-431542 down-regulate intracellular SMAD3 expression 35,56 to induce C/EBPα activation. Additionally, type B vitamins such as biotin 29,41,57,58, pantothenate 29,41,57,58, and apo-transferrin 58 and type C vitamins 40,53,57,59,60 are usually supplemented.

For browning, PRDM16 plays a pivotal role in driving the thermogenic program 24 with the assistance of EBF2 in the early stage 39 and PGC-1α to co-activate several transcriptional factors 61. Besides, PPARγ also contributes to the thermogenic program by increasing the half-time of PRDM16 24,62. T3 29,41,63 and rosiglitazone 36,63-66 mentioned above are common browning compounds. Particularly, T3 promotes browning by supporting BAP proliferation 67 and stimulating PGC-1α 68 and UCP1 39,63,69-71. TGF-β inhibitors 36 also promote browning by activating EBF2 39. Besides, BMP7 activates PRDM16 37 and promotes mitochondrial biogenesis by activating the p38 MAPK signaling pathway 40,64. Additionally, ROCK inhibitors such as Y-27632 64 and transferrin 65,72 are also sometimes included in the browning medium. β-adrenergic agonists such as mirabegron and CL316,243 and cAMP activators such as forskolin, which directly mimic external β-adrenergic stimulation in vivo, also contribute to the generation of BrAOs and BeAOs in vitro 34,73.

### 3.2 Sources for adipose organoids

#### 3.2.1 PSCs

Previously, researchers differentiated PSCs into embryonic bodies (EBs) in suspension and derived a mixture of MSCs after transferring them to adherent plates. Retinoic acid pretreatment combined with CD73 selection 53,74 or PPARγ transduction 48 generated HOXC8/9^+^ HOXA5^+^ BMP4^+^ white adipocyte progenitors. Meanwhile, CD73 selection without retinoic acid pretreatment 53,74 or PRDM16 75, PPARγ2-CEB/Pβ 48, or PPARγ2-CEB/Pβ-PRDM16 48 transduction enabled PAX3^+^ CIDEA^+^ CD137^+^ brown adipocyte progenitors. The derived white and brown adipocyte progenitors differentiated into white and brown adipocytes, respectively.

However, these strategies did not undergo defined mesoderm stages, thus causing the identity of derived brown adipocytes to be questioned 35. Based on current knowledge about adipose tissue development, several studies have designed stepwise methods to derive beige and brown adipocytes 57. BMP4 plays an essential role in the commitment of SplM from PSC to derive beige adipocytes 39,60. Guénantin et al. induced hiPSCs to PDGFRA^+^ LY6E^+^ CD29^+^ beige adipocyte progenitors in a hematopoietic medium containing BMP4 and Activin A^65^. Besides, Su et al. induced hiPSCs to SplM by activating the BMP, WNT, and VEGF signaling pathways 39. They overcame the adipogenic differentiation limitation of FOXF1^+^ SplM-derived MSCs by inhibiting TGF-β and activating IL-4. The derived beige preadipocytes differentiated into beige adipocytes in the adipogenic differentiation medium supplemented with browning compounds 39. These SplM-derived beige adipocytes exhibited high-level and β-adrenergic responsive OCR 39. They also secreted anti-T2DM adipokines to improve the glucose metabolism of white adipocytes from T2DM patients 39,60.

Activation of WNT and FGF signaling pathways, along with BMP inhibition, are crucial for the commitment of paraxial mesoderm from PSCs to obtain brown adipocytes 35,57. Zhang et al. induced hiPSCs to PAX3^+^ MYF5^+^ FOXC1^+^ paraxial mesoderm by activating WNT and FGF while inhibiting the BMP signaling pathway. Paraxial mesoderm eventually differentiated into mature brown adipocytes with high-level and responsive glycolysis, lipolysis, and OCR. Besides, they increased thermogenesis and energy expenditure, and improved glucose hemostasis of hyperglycemia mice after transplantation 35. Similarly, Carobbio et al. induced MYF5^+^ PDGFRα^+^ paraxial mesoderm from hESCs by activating WNT, FGF, and retinoic acid signaling pathways and then to brown adipocyte precursors with BMP inhibitors such as LDN-193189 supplemented. They eventually derived KCNK3^+^ MTUS1^+^ ITGA10^+^ multilocular brown adipocytes after adipogenic differentiation 57.

#### 3.2.2 Cells derived from adipose tissues

Cells derived from adipose tissues capable of the generation of adipose organoids include ADSCs, SVF-WATs, and SVF-BATs. ADSCs are commonly used for WAOs while browning molecules 64,65,72 are necessary for white-to-beige transition 27,31,64,65,72,76. Singh et al. realized the beige differentiation efficiency of ADSCs to over 90% by supplementing T3, rosiglitazone, BMP-7, and Y-27632 in adipogenic differentiation medium 64. Beige adipocytes exhibited higher levels of cAMP-sensitive uncoupled respiration, glycolysis, and lipolysis compared with white adipocytes 64. SVFs contain heterogeneous subpopulations of cells, including endogenous ECs and immune cells 7, thus showing advantages in vascularization and modeling inflammation. Immortalization of SVFs clearly described in articles 34,63 is necessary for application. The fate of SVFs is predetermined; therefore, basal adipogenic differentiation medium supplemented with T3 is sufficient for the differentiation of SVF-WATs and SVF-BATs 29,41,63. Browning molecules can enhance the differentiation efficiency of SVF-BATs 36 but have limited impact on promoting the white-to-beige transition of SVF-WATs 41. With T3 and rosiglitazone supplemented to augment browning efficiency, brown adipocytes differentiated from SVF-BATs exhibited higher levels of maximum and uncoupled OCRs in both basal and cAMP-stimulated states, glucose uptake in basal and insulin-stimulated states, as well as fatty acid uptake and oxidation 41,63. Wang et al. successfully realized the white-to-beige transition of SVF-WATs by activating endogenous UCP1 using CRISPR-Cas9. Such brown-like adipocytes exhibited higher levels of glucose uptake, glucose-dependent and fatty acid-dependent uncoupled OCRs, and thermogenic capacity compared with white adipocytes 77.

#### 3.2.3 Adipose tissues

Micro adipose tissues maintain complex cellular and ECM components for a fidelity microenvironment in adipose organoids 28. Micro-WATs are usually obtained by cutting subcutaneous adipose tissues (SATs) into small pieces (0.5-5 mm) 34,78, mincing through 19-gauge needles, or liquefying in a blender through continuous short pulses. Through embedding fragments of the rat superficial fascia (1-3 mm^3^) in fibrin hydrogels, seeding liquified human lipoaspirates into silk scaffolds, or sandwiching human primary WAT fragments (0.5-1 mm) between SVF-derived adipose sheets, WATs were reconstructed to maintain viable and functional for longer time (over 3 months) 28,79 than primary explants. These WAOs exhibited unilocular structure and functions in lipogenesis 28,44, lipolysis 28,44,79, and adipokine secretion 28,44,79. To generate BeAOs from micro-WATs, β-adrenergic agonists, cAMP activators, and angiogenic compounds are usually supplemented. Blumenfeld et al. directly converted both SAT and visceral adipose tissue (VAT) fragments into 'brown' adipose organoids in adipogenic differentiation medium supplemented with T3, rosiglitazone, CL316,243, and VEGF for 3 weeks. The organoids contained multilocular adipocytes with high UCP1 expression and mitochondrial metabolic activity. They maintained the thermogenic phenotype for at least 8 weeks after transplantation 73. However, considering the SplM origin, such 'brown' adipose organoids might theoretically be BeAOs which requires further validation.

### 3.3 3D techniques for adipose organoids

**Figure 2:**
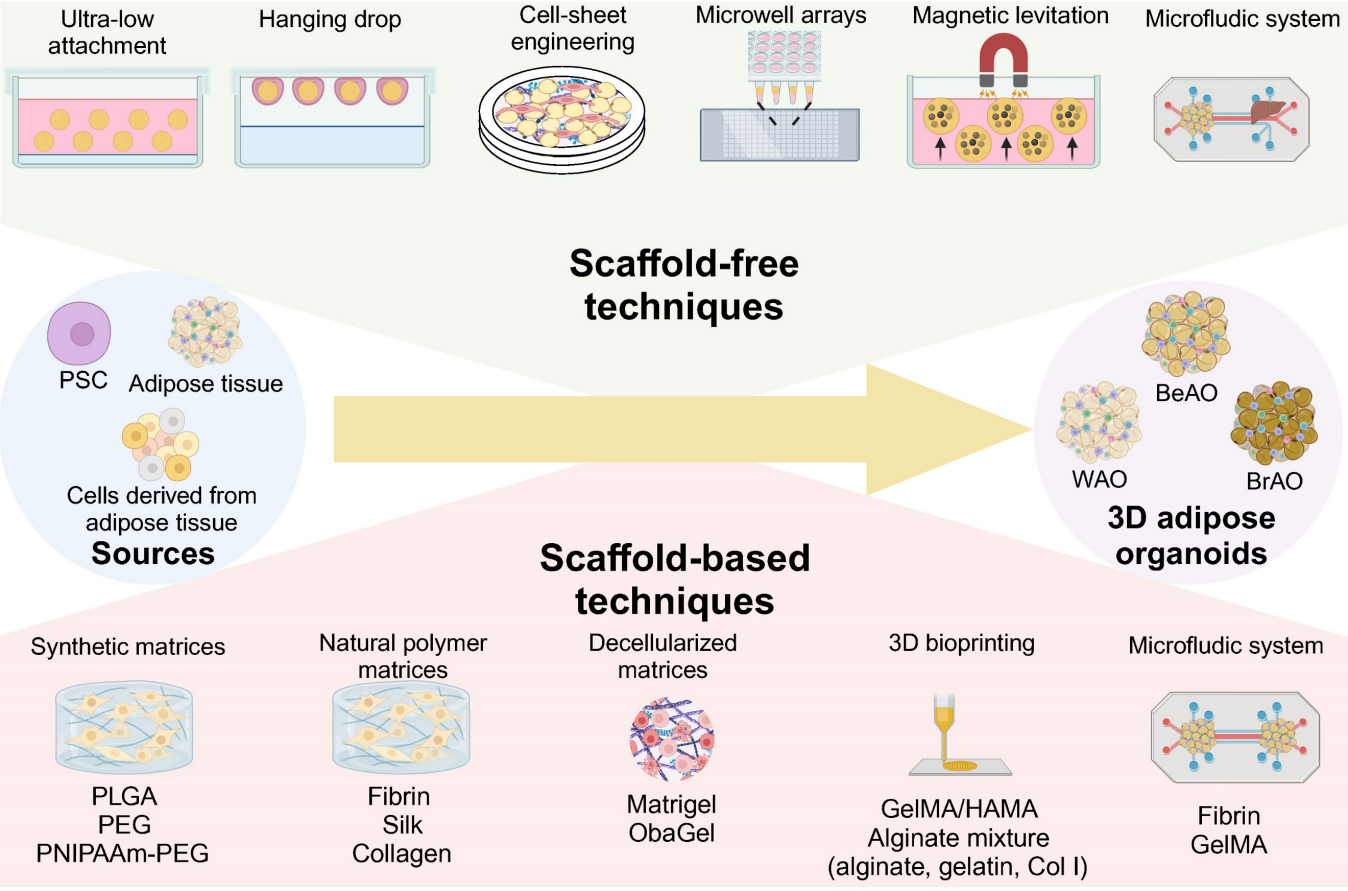
** Scaffold-free and scaffold-based strategies to establish 3D adipose organoids.** Through three-dimensional (3D) techniques including scaffold-free and scaffold-based techniques, 3D adipose organoids can be fabricated from various sources. The ultra-low attachment technique is the most widely used scaffold-free technique due to its automation, simplicity, and high throughput. The hanging drop technique enables precise size control, but is challenging to replenish the low-volume culture medium. Cell-sheet engineering enables the generation of adipose organoids through superposition. Microwell arrays perform well in precisely controlling the diameters of adipose organoids by limiting seeded cell numbers. The magnetic levitation technique also enables the establishment of adipose organoids composed of more than one cellular component through positive or negative magnetophoresis. The microfluidic device provides a chance to recapitulate the communication between adipose tissues and other organs. Scaffold-based techniques include synthetic, natural polymer, and decellularized matrices that can be used alone or in combination with 3D bioprinting and microfluidic systems. Scaffolds promote cell attachment and migration and inhibit excessive cell aggregation. They also promote sprouting, adipogenesis, maintenance of depot-specific and subject-specific characteristics, and long-term preservation of viability and function of adipose organoids. BeAO: beige adipose organoid; BrAO: brown adipose organoid; Col I: type I collagen; dECM: decellularized extracellular matrix; GelMA: methacrylate gelatin; HAMA: hyaluronic acid; PEG: poly(ethylene-glycol); PLGA: poly(lactic-co-glycolic acid); PNIPAAm: poly(N-isopropylacrylamide); PSC: pluripotent stem cells; WAO: white adipose organoid; 3D: three dimensional.

In addition, the role of the 3D microenvironment for adipose organoids is also unneglected. Compared with traditional 2D culture, 3D shows the following advantages: 1) 3D maintains depot-specific characteristics^80^; 2) 3D increases cell-cell adhesion and crosstalk 80; 3) 3D links the organotypic microenvironment to improve adipogenesis 26,81-84; 4) 3D is more responsive to insulin and pro-inflammatory stimuli and sensitive to metabolic and environmental stress 26,85,86; 5) 3D promotes stemness and long-term survival 82,85; 6) 3D is feasible to manipulate biochemical, mechanical, topographic, and cellular microenvironment 29,85; 7) 3D secrets higher levels of adipokines such as adiponectin 26. Current 3D techniques for adipose organoids are summarized in **Figure 2**.

#### 3.3.1 Scaffold-free techniques

For scaffold-free techniques, cells are exposed to a non-adherent environment to attach to each other, synthesize and remodel their own ECM, and finally form a 3D agglomerate through cell-cell and cell-matrix interaction. For the ultra-low attachment technique, ADSCs 87,88, SVFs 81,89-91, or predipocytes 86 were seeded in ultra-low attachment microwells in static culture 81,86,87,89,90,92, or dynamic culture involving stirring 91 or shaking 88. It also enabled the establishment of disease models from ADSCs of lipedema patients 88 and perinephric adipose tissue SVFs of kidney tumor patients 89. Notably, Turner et al. designed elastin-like polypeptide (ELP)-polyethyleneimine (PEI)-coated 24-well plates to establish 45 μm adipose spheroids by seeding 50,000 3T3-L1 preadipocytes 86 or ADSCs 92 per well. The ELP promotes spheroid formation, and PEI promotes spheroid attachment to the surface. Such 3D spheroids outperformed the 2D monolayer adipocytes in responsiveness to fatty acid exposure and TNF-α stimuli for obesity modeling 86,92. Through the hanging drop method, preadipocytes 93, SVFs 26, ADSCs 58, or microvascular fragments (MVFs) 94 self-assembled due to gravity. The cell suspension of 20-28μl per droplet was placed on the inside lid of the plate and inversed for 2-5 days to form spheroids due to gravity 26,58,93,94. Quan et al. generated well-vascularized adipose organoids (300-400 μm in diameter) exhibiting responsive lipolysis and adipokine secretion 94. Adipose organoids (150 μm in thickness) were also reconstructed via the superposition of three adipose sheets derived from ADSCs in adipogenic differentiation medium supplemented with Vitamin C 27,79,95-97. Adipose organoids remained viable for at least 11 weeks and secreted higher levels of leptin, plasminogen activator inhibitor-1, and angiopoietin-1 than primary fat explants 97.

Considering the high demand for glucose, oxygen, and nutrients of thermogenic adipocytes, precise control of the diameter of BrAOs or BeAOs is crucial^36^. Microwell arrays perform well in the production of size-controlled organoids. Oka et al. generated adipose spheroids (100-120 μm in diameter) by seeding hPSCs on the Elplasia® 3D discovery tool with 90 microwells per well to restrict cell numbers within a range of 2,000-2,500 cells for each spheroid formation 98. They derived BrAOs containing multilocular adipocytes with dense mitochondria in the aforementioned hematopoietic medium 98. These BrAOs with optimal diameters secreted batokines which promote insulin secretion of β cells and maintained thermogenic capacity after transplantation 98. For the magnetic levitation technique, mice SVFs 99 or preadipocytes 100 were mixed with nanoshuttles to be magnetically levitated in positive magnetophoresis for aggregation 99,100. The derived WAOs recapitulated the molecular signaling pathway of WAT organogenesis 99,100 and complex vascularized structures through co-culture with ECs 100. However, unremovable nanoshuttles may induce DNA cytotoxicity once internalized 101,102. Sarigil et al. first demonstrated the utilization of the negative magnetophoresis for WAO establishment from adipocytes and even multilayered ones by co-culturing with bone marrow-derived mesenchymal stem cells. This label-free method holds potential for clinical regenerative medicine if further function validation can be conducted 103. Microfluidic devices supported complete on-chip differentiation of human preadipocytes to form WAOs responsive to proinflammatory, obese, and diabetic conditions. This organ-on-chip technology successfully recapitulated communication between adipose tissues and livers 104.

#### 3.3.2 Scaffold-based techniques

Besides, other strategies utilized scaffolds containing ECM or ECM-like components for the 3D adipose organoid formation via cell-matrix interaction. Common scaffolds including synthetic, natural polymer, and decellularized matrices promote cell attachment and migration while inhibiting excessive cell aggregation. The physicochemical environment of matrices also affects white and brown adipogenesis, maintenance of depot-specific characteristics, and metabolic functions 29,36,105,106. However, the safety and ethical concerns of synthetic and animal-derived hydrogels hinder their further clinical application. Therefore, thermoreversible poly(N-isopropylacrylamide)-poly(ethylene-glycol) (PNIPAAm-PEG) hydrogels and human-derived decellularized ECMs (dECMs) have been designed for better bench-to-bedside translation 36,107.

Synthetic matrices commonly include porous poly(lactic-co-glycolic acid) (PLGA) hydrogels and PEG-based hydrogels. The porous PLGA hydrogels promoted the generation of size-controlled WAOs (220 μm in diameter) with angiogenic adipokine secretion 76. PEG-based hydrogels are easy to modify, like with RGD-containing peptides to further promote initial cell attachment and adipogenic differentiation^106^. Besides, Wang et al. designed thermoreversible PNIPAAm-PEG hydrogels to form size-controlled BrAOs with high yield (1 x 10^7^ cells/ml hydrogel). PNIPAAm-PEG hydrogels enabled ideal short-term preservation at room temperature and long-term cryopreservation without sacrificing viability and could be easily removed before transplantation 36.

For natural polymer matrices, fibrin hydrogels promoted superficial fascia fragments 44, ADSCs 25,31,108, and MVFs 38 to form WAOs containing unilocular adipocytes with lipolysis and adipokine secretion functions 25,31,44,108. Thin fibrin-based cellulose scaffolds further supported the assembly of WAOs sensitive to fatty acid exposure to model obesity 25. By seeding human liquified lipoaspirates in silk scaffolds, fragile WATs can be endocrinologically functional for the long term. Considering the availability of a small volume of lipoaspirates, this method holds potential for studying patient-specific drug response and pathogenesis 28,78. Meanwhile, fibrin promoted the maintenance of subject-specific differences between lean and T2DM patients 38, while collagen supported the preservation of depot-specific characteristics 105.

For decellularized matrices, Matrigels facilitate the sprouting of SVFs and micro-WATs to derive beige adipocyte progenitors in angiogenic endothelial growth medium (EGM) 34,91. To address the ethical concerns, human blood-derived ObaGels were designed to generate human SVF-derived WAOs capable of lipolysis, glucose uptake, and leptin secretion 107. In particular, the proteomic profile and physical microenvironment of dECMs show differences between obese and lean donors, with a pro-inflammatory microenvironment in the former 109. Therefore, subject-specific dECMs should be further applied to patient-specific adipose organoid generation 110.

Besides, methacrylate gelatin and hyaluronic acid (GelMA/HAMA) hydrogels and alginate hydrogel mixture are commonly employed as bioinks for 3D bioprinting. GelMA/HAMA hydrogels enabled the study of optimal mechanical conditions for different adipocyte adipogenesis. It was found that soft solid hydrogels efficiently promoted white adipogenesis, whereas stiff porous hydrogels promoted brown adipogenesis 29. 3D-printed BrAOs in the alginate hydrogel mixture exhibited high UCP1 expression 111, while WAOs mimicked insulin-resistance through macrophage co-culture for disease modeling and drug screening of T2DM 83,84,112. Paek et al. designed a hybrid microfluidic platform containing perfusable microchannels in a fibrin hydrogel to establish well-vascularized WAOs with ideal leptin secretion 31. Besides, by embedding ADSCs from infrapatellar fat pads of osteoarthritis patients, O'Donnell et al. first cultured patient-specific WAOs with robust viability and adipogenesis in both static and dynamic conditions in a perfusion bioreactor to study the role of infrapatellar fat pads in osteoarthritis progression 113.

### 3.4 Strategies to improve adipose organoid maturation

Vascularization and the incorporation of immune cells can improve the maturity of adipose organoids. Vascularization promotes adipogenesis and browning, as well as facilitates integration with host vessels for long-term maintenance of implants 36,80. In detail, adipocytes near vessels exhibit superior lipid accumulation 114. Meanwhile, angiogenic cocktails promote beige preadipocyte proliferation and enhance brown differentiation efficiency 29,34,38,40,53. Therefore, more studies have focused on the vascularization of BeAOs and BrAOs. Through co-culture with macrophages, WAOs mimicked the mild and chronic inflammation state and the progression of insulin-resistance in T2DM. However, both the incorporation of macrophages into BeAOs and BrAOs and other immune cells into adipose organoids to better recapitulate cell crosstalk are nascent study areas that need to be further investigated 83,84,112,115.

#### 3.4.1 Promoting vascularization

The incorporation of exogenous or endogenous ECs promoted the vascularization of adipose organoids. Through pre-exposing human co-cultured ADSCs and ECs or BAT-SVFs to angiogenic EGM before adipogenic induction, adipose organoids were well vascularized 31,36 and innerved after transplantation and showed therapeutic effects on obese and T2DM mice 36. For MVFs, pre-exposure to growth medium just promoted vasculariztion 38. Besides, adipose sheets co-cultured with ECs were vascularized with unimpaired leptin secretion function through post-exposure to angiogenic EGM at the later stage of adipogenic differentiation 96,97. Moreover, the all-in-one medium by mixing adipogenic differentiation medium with EGM in a 1:1 ratio also promoted the generation of vascularized adipose organoids responsive to hyperinsulinemia from co-cultured ADSCs and ECs 116.

**Figure 3:**
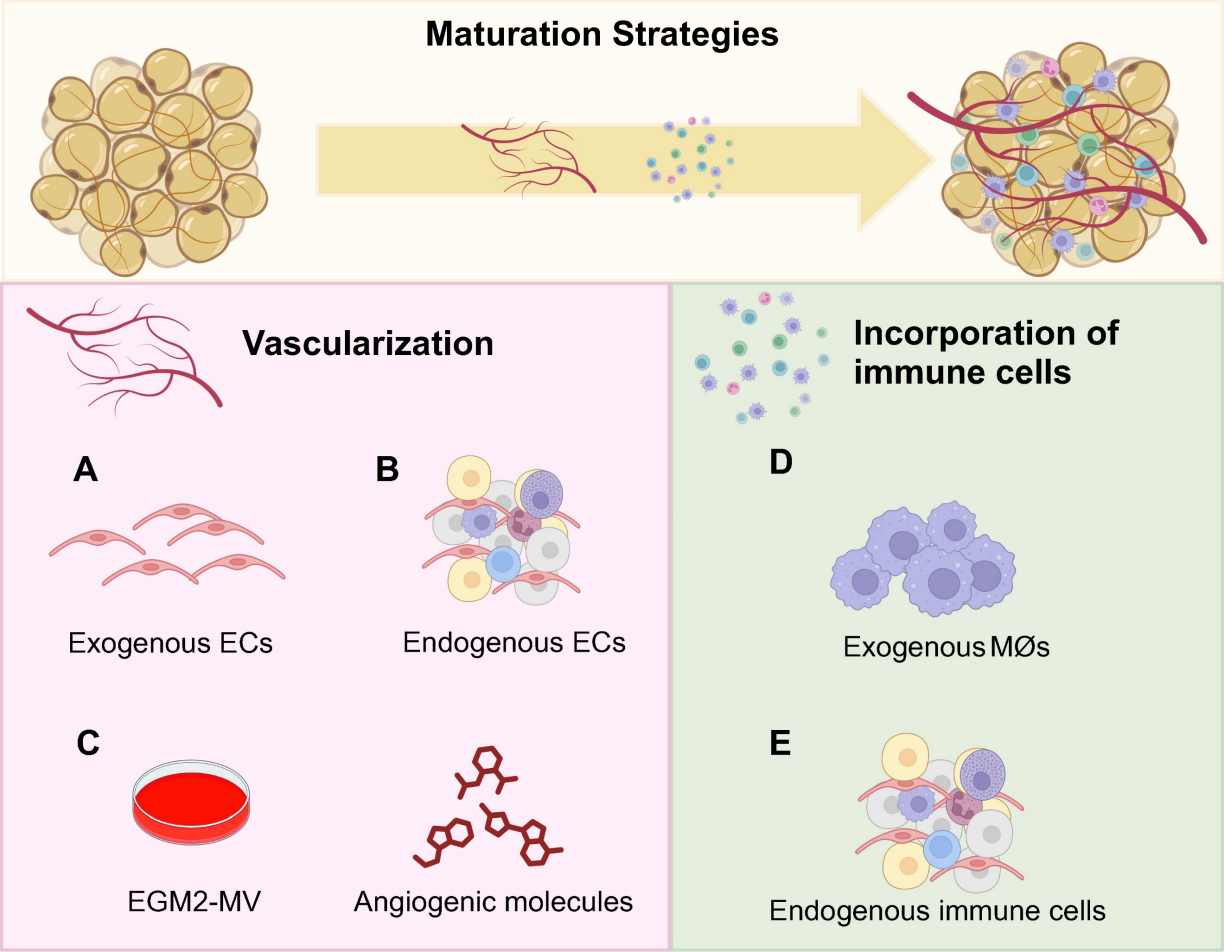
** Promoting vascularization and incorporating immune cells to facilitate maturation of adipose organoids.** Considering the native adipose tissue niche, both vascularization and immune cell incorporation are crucial for the maturation of adipose organoids. Regarding research intensity, vascularization has been predominantly investigated in brown adipose organoids and beige adipose organoids to promote browning. Although incorporating immune cells is essential for all types of adipose organoids, currently only mice macrophages were successfully incorporated into white adipose organoids for disease modeling and drug screening. No studies have generated brown or beige adipose organoids with macrophages incorporated yet. By incorporating exogenous human microvascular endothelial cells and human umbilical vein endothelial cells (a), or using stromal vascular fragments, microvascular fragments, and adipose tissue explants with endogenous endothelial cells (b), well-vascularized adipose organoids can be successfully generated. (c) The supplementation of angiogenic compounds such as VEGF, IL-6, KITLG, and FLT3LG, along with endothelial growth medium further promotes vascularization, beige preadipocyte proliferation, and ex vivo browning. (d) By co-culturing mice macrophages, RAW264.7, with mice 3T3-L1 preadipocytes or human adipose-derived stem cells in alginate hydrogel mixture by three-dimensional bioprinting, inflamed white adipose organoids with insulin-resistance were successfully generated. (e) By directly inducing the adipogenic differentiation of stromal vascular fragments, adipose organoids with resident CD45^+^ CD31^-^ immune cells preserved such as macrophages and mast cells were generated. BeAO: beige adipose organoid; BrAO: brown adipose organoid; EC: endothelial cell; EGM2-MV: endothelial cell growth medium 2-microvascular; Mø: macrophage; WAO: white adipose organoid.

Notably, vascularization exerts an active effect on thermogenic programs by activating PRDM16 and UCP1 via the p38 MAPK signaling pathway 40,59. Pre-exposure to EGM promoted beige preadipocyte proliferation around sprouting vessels from micro-WATs embedded in Matrigels 34. Moreover, hematopoietic cocktails (IGF-II, VEGFA, KITLG, FLT3LG, and IL-6) supplemented with BMP4 and then BMP7 directly induced hiPSCs into brown adipocytes with high-level and responsive UCP1 expression and OCR 40. The brown adipocytes promoted lipid and glucose metabolic hemostasis after transplantation and secreted hematopoietic adipokines potential for myelosuppression treatment 40. Size-controlled BrAOs, further established in microwell arrays using the same hematopoietic recipes, secreted batokines capable of augmenting the insulin secretion of β cells (**Figures 3A-C**) 98. Notably, VEGFA 73 and retinoic acid 59 also promoted the browning of adipose organoids through increasing vascularity by activating VEGFA/VEGFR2 signaling 59.

#### 3.4.2 Incorporating immune cells

WAOs incorporated with macrophages better mimicked insulin-resistance compared with monocultured ones validated by proteomic analysis 112. In addition to co-culturing mice 3T3-L1-derived adipose organoids with mice macrophages, by loading mice macrophages and human ADSC-derived WAOs in designed separate wells with interaction preserved, errors resulting from interspecies variation can be minimized for further preclinical research (**Figure 3D**) 83,84. Furthermore, direct induction of SVFs shows the potential to solve the species issue by preserving resident immune cells in adipose organoids. After adipogenic differentiation without any additional immune cell growth factors supplemented, CD45^+^CD31^-^ immune cells in mice SVFs were maintained, which can be attributed partly to the abundant macrophage colony-stimulating factors secreted from undifferentiated adipose organoids. Among the immune cell populations, 60-70% were CD11b^+^F4/80^+^ macrophages, while approximately 10% were FcεR1^+^ckit^+^ mast cells. Therefore, SVF-derived WAOs serve as an ideal platform for modeling inflammatory states and investigating cell crosstalk between immune cells and adipocytes (**Figure 3E**).

Human SVFs should be further applied to the establishment of human adipose organoids with immune cells retained. Currently, only monocytes and macrophages have been incorporated in WAOs. However, in vivo, eosinophils and Tregs secrete factors that induce M2 polarization for adipose tissue homeostasis, while neutrophils, CD8^+^ cells, NK cells, Th1 cells, and B2 cells induce M1 polarization in hypertrophic adipose tissues 23,117. Eosinophils and M2 also enhance the activity of brown and beige adipocytes 24,118. Therefore, subsequent studies should attempt to incorporate these immune cells into adipose organoids.

## 4. Applications of adipose organoids in obesity-related metabolic diseases

### 4.1 Obesity

As previously mentioned, obesity caused by excessive energy uptake is characterized by adipose tissue inflammation. Current treatment strategies encompass lifestyle intervention which is challenging to keep, complex medications associated with adverse gastrointestinal or neuropsychiatric effects, and bariatric surgery with complications such as cholelithiasis 12. Therefore, through lipid exposure, pro-inflammatory stimuli, or co-culture with macrophages, WAOs are capable of disease modeling and drug screening of obesity to identify novel therapies with high safety and efficacy. Moreover, BrAO and BeAO transplantation can realize long-term and stable weight loss primarily by increasing energy expenditure.

Obesity was successfully modeled by exposing WAOs to fatty acids. Extra saturated palmitic or stearic acids, unsaturated oleic acids, or a natural lipid mixture induced an increase in droplet sizes and basal lipolysis 25,86,119,120, correlated with an increase in circulatory levels of fatty acid and glycerol in vivo 86,120. Fatty acid exposure also induced insulin-resistance and pro-inflammatory cytokine secretion 25,120. Moreover, the ratio of adiponectin to leptin secretion was reduced, which indicates adipose tissue dysfunction and the development of a pro-inflammatory obesogenic state 120. Pieters et al. embedded ADSCs in fibrin/Geltrex™ hydrogels and infiltrated them into thin cellulose scaffolds to efficiently assemble WAOs. Exposure to fatty acids effectively enabled obesity modeling with increased lipid droplet sizes and induced insulin-resistance. Specifically, palmitic acids instead of oleic acids increased basal lipolysis and secretion of pro-inflammatory cytokines which alter macrophage gene expression 25.

Another approach for obesity modeling is to expose WAOs to TNF-α or co-culture them with macrophages under lipopolysaccharide (LPS) stimuli. TNF-α and IL-1β treatment resulted in an increase in pro-inflammatory cytokine secretion and a detrimental effect on the microvascular network in vascularized adipose sheets 95. Abbott et al. established WAOs by seeding small volumes of liquified lipoaspirates from different individuals into silk scaffolds, enabling assessment of individual responses to TNF-α and serving as a proof-of-concept for future patient-specific preclinical studies 28. Surprisingly, there were no significant differences in glucose uptake and lipid accumulation under TNF-α stimuli among WAOs derived from subjects with different body mass indexs 78.

**Figure 4:**
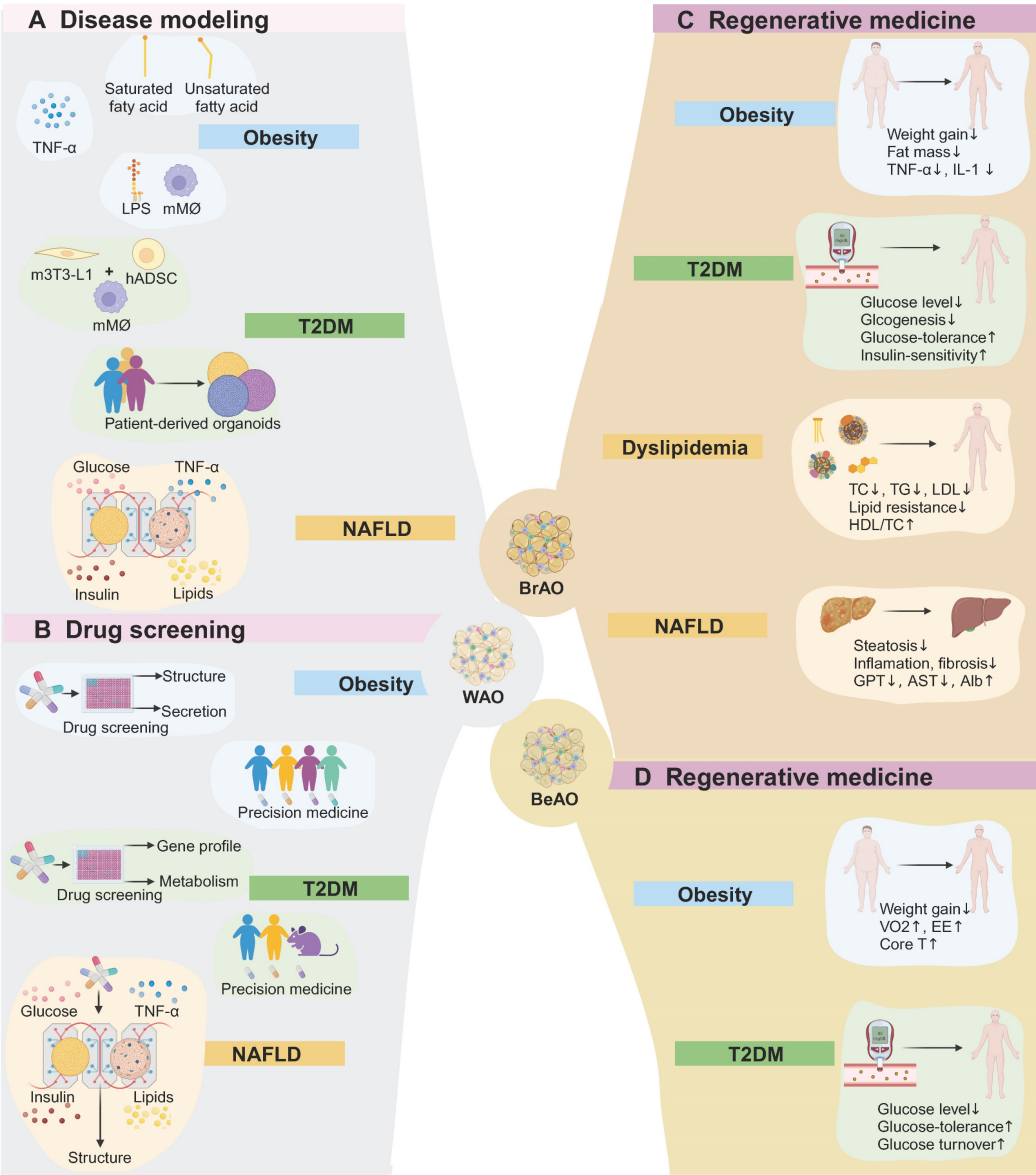
** Application of adipose organoids in disease modeling, drug screening, and regenerative medicine.** (a) White adipose organoids can model obesity through exposure to fatty acids or TNF-α, or co-culture with immune cells under lipopolysaccharide stimuli. Disease modeling of type 2 diabetes mellitus (T2DM) can be established through co-culture adipose organoids with macrophages, or from patient-derived adipose organoids. Besides, a microfluid system is applied to investigate the role of adipose tissues in the progression of non-alcoholic fatty liver disease (NAFLD) by mimicking healthy, obese, diabetic, and pro-inflammatory plasma conditions. (b) By assessing the effects of compounds on lipid accumulation, adipogenic gene expression, pro-inflammatory cytokine secretion, and metabolism such as glucose uptake, white adipose organoids can be applied to high throughput drug screening and personalized precision medicine in obeisity, T2DM, and NAFLD. (c) Brown adipose organoids reduce body weight and fat mass as well as relieve inflammation after transplantation. Transplantation also shows therapeutic potential in T2DM by reducing glucose levels and improving glucose intolerance and insulin-resistance. Besides, it regulates dyslipidemia, relieves inflammation and fibrosis in livers, and improves liver function. (d) Beige adipose organoid transplantation shows potential for treating obesity. Additionally, it also reduces glucose levels and improves glucose tolerance and turnover. Alb: albumin; AST: aspartate transaminase; BeAO: beige adipose organoid; BrAO: brown adipose organoid; Core T: core temperature; EE: energy expenditure; GPT: glutamate pyruvate transaminase; hADSC: human adipose-derived stem cells; HDL/TC: the ration of high-density lipoprotein to total cholesterol; LDL: low-density lipoprotein; LPS: lipopolysaccharide; mMø: mice macrophage; m3T3-L1: mice preadipocytes; NAFLD: non-alcoholic fatty liver disease; TC: total cholesterol; TG: triglyceride; T2DM: type 2 diabetes mellitus; VO2: maximum volume of oxygen; WAO: white adipose organoid.

The incorporation of immune cells under LPS stimuli also successfully induced inflammation of WAOs. Harms et al. designed membrane mature adipocyte aggregate cultures to maintain depot-specific characteristics of mature adipocytes for long-term culture and placed macrophages on top of the membrane under LPS treatment. A higher increase in IL-6 and IL-8 secretion of adipocytes and TNF-α and IL-6 secretion of macrophages was induced than in mono-culture 43. For SVF-derived WAOs with resident immune cells, LPS stimuli also significantly increased the secretion of IL-6 and the obesity-induced chemokine C-C Motif Chemokine Ligand 2 as well as induced a decrease in diacylglycerol and an increase in cholesterol (**Figure 4A**) 119. Besides, WAOs can further mimic long-term chronic inflammation of adipose tissue through FGF2 exposure to induce fibrosis (**Table 1**) 121.

Adipose organoids can also be applied to high-throughput screening of anti-obesity drugs. Choi et al. formed WAOs by co-culturing mice preadipocytes and macrophages in alginate mixture hydrogel beads. This 3D co-culture system served as a successful high-throughput screening platform validated by its sensitive and dose-dependent responses to rosiglitazone and GW9662 compared with 2D co-culture. Using AdipoRed/dsDNA to evaluate the percentage of adipogenesis inhibition as the first hit, seven compounds exhibiting opposite effects in 3D versus 2D were selected among 178 compounds consisting of adenosine monophosphate-activated protein kinase activators, PPARγ agonists, and PPARγ antagonists. Then, three compounds exhibiting repetitive anti-adipogenic effects in 3D were selected by investigating IC_50_ values 122. Besides, the easily generated WAOs derived from liquefied lipoaspirates show promise in personalized medicine, as evidenced by their patient-specific responses to AICAR 78. Combined with high-content confocal and epifluorescence analysis to distinguish well-differentiated spheroids from undifferentiated ones, human SVF-derived WAOs also show potential for high-throughput screening of anti-obesity drugs (**Figure 4B** and **Table 2**) 26.

Transplantation of brown 65 and beige adipocytes 64 as well as BrAOs 36,73 reduced body weight 36,64,65,73 and fat mass 36,73, showing significant less weight and fat mass gains compared to the control group with hADSC 65,123 or white fat explants 73 being transplanted or the sham group 36 2-26 weeks after transplantation. These results were attributed to the significantly enhanced energy expenditure resulting from the non-shivering thermogenesis of transplanted thermogenic adipocytes. Specifically, a significantly higher value of volume of oxygen, energy expenditure, as well as core and surface temperature compared to the control group were simultaneously observed post-transplantation 64. Meanwhile, as mentioned before, obesity is characterized by a state of low-grade and systematic inflammation.

Correspondingly, the transplantation of thermogenic adipocytes resulted in the alleviation of inflammation, evidenced by significantly reduced levels of TNF-α and IL-1 expression in adipose tissues and an increased ratio of adiponectin to leptin 65. The host models included obese C57BL/6 mice fed with high-fat diet (HFD) 65,73, hyperglycemia non-obese diabetic/severe combined immunodeficiency (NOD/SCID) mice injected with streptozotocin (STZ) 64, and T2DM Rag1 ^-^/^-^ mice injected with STZ after 3-month HFD 36. The ideal transplantation region encompassed hindlimb muscle 64,73 and kidney capsule 36 which provide a pro-browning microenvironment to avoid whitening. Wang et al. injected human BrAOs (with 1.25 million adipocytes) derived from immortalized SVF-BATs into the kidney capsule of Rag1 ^-^/^-^ mice. After transplantation for 18 days, mice were fed with 3 months of HFD and injected with low-dose STZ (90 mg/kg) to establish obesity and T2DM models. Compared with the sham group, the transplantation group exhibited significant suppression in body weight and fat mass gains with UCP1 expression maintained in implants after transplantation for 5 months 36. Besides, autologous reimplantation of BrAOs (0.2-0.3 grams) directly converted from SATs exhibited persistent brown-like phenotype after transplantation for 8 weeks, but its anti-obesity effect needs further evaluation (**Figs. 4C-D** and **Table 3**) 73.

### 4.2 Type 2 diabetes mellitus

According to the International Diabetes Federation in 2021, approximately 537 million people worldwide have diabetes, with 90% having T2DM 124. The extensive prevalence of T2DM has positioned it as one of the most significant public health challenges 124,125. Obesity stands out as a crucial risk factor for T2DM 12,18,125. Mechanistically, hypertrophic adipose tissues release pro-inflammatory cytokines and toxic lipolysis products that usually induce insulin-resistance in adipose tissues, livers, and skeletal muscles by inhibiting the IRS1-AKT-GLUT4 signaling pathway 32,117,126,127. The prediabetic condition gradually progresses to T2DM as compensation for insulin-resistance diminishes 128,129. Common T2DM therapies include dietary and lifestyle interventions along with medications 13,129,130. However, anti-diabetes drugs may cause adverse effects such as hypoglycemia, weight gain, and oedema 13,131. For the discovery of innovative therapies targeting T2DM, WAOs co-cultured with macrophages are employed to mimic insulin-resistance for disease modeling and drug screening. Transplantation of BrAOs and BeAOs holds promise in treating T2DM by ameliorating glucose levels and improving insulin-resistance and glucose intolerance.

WAOs were co-cultured with mice macrophages in the alginate hydrogel mixture without impairing the adipogenic differentiation capacity to model insulin-resistance 83,84,112,122, characterized by lower p-AKT and GLUT4 expressions 83,84,122 and insulin-stimulated glucose uptake (ISGU) level^94,95,12^ than in the mono-culture system. Park et al. inflamed WAOs by mixing mice preadipocytes and macrophages in the alginate hydrogel mixture. Macrophages exhibited enhanced proliferation and activation in adipogenic differentiation medium, as evidenced by increased expression of pro-inflammatory TNF-α and IL-1β, F4/80, and cytochrome C. The macrophage co-culture attenuated GLUT4 and pAKT expressions, the membrane-to-cytosol expression ratio of GLUT4, and ISGU 83. Acosta et al. established patient-derived adipose organoids by seeding MVFs in fibrin hydrogels. The T2DM-derived adipose organoids showed less angiogenic capacity, but comparative lipolytic level and thermogenic potential relative to adipose organoids from lean mice (**Figure 4A** and **Table 1**) 38.

The co-culture system also served as a high-throughput screening platform for anti-T2DM drugs 83,84, validated by commercial drugs including rosiglitazone 83, and/or acarbose, metformin, and exendin-4 84. Park et al. assessed the effects of five PPARγ antagonists on altering GLUT4 and p-AKT expressions and glucose-6-phosphate dehydrogenase activity. GW9662 exhibited optimal performance in improving insulin-resistance and enhancing glucose uptake 83. However, interspecies differences in drug effects between mice-derived WAOs and human adipose tissues impede the clinical translation of screened drugs. Therefore, they further designed separate wells with cell interaction maintained to load human or mouse WAOs with mice macrophages to minimize interspecies errors. They employed 11β-HSD1 inhibitors, including KR-1 (mouse-potent enantiomer), KR-2 (human-potent enantiomer), and KR-3 (racemic compound) for validation. As a result, KR-2 and KR-3 better ameliorated insulin-resistance in human ADSC-derived WAOs, whereas KR-1 and KR-3 exhibited better effects on mice 3T3-L1-derived WAOs (**Figure 4B** and **Table 2**) 84.

Transplantation of beige and brown adipocytes as well as BrAOs decreased the glucose level 34-36,64-66,75 as well as improved glucose intolerance 34-36,64-66,75,77 and insulin-resistance 36,65,75,77. Beige adipocytes 34 and brown adipocytes 65,66,75,77 were transplanted to obese C57B/L6 65,75, NOD-*scid IL2rγ^null^* (NSG) 34,66 or BALB/c athymic nude mice fed with HFD 77. Besides, beige adipocytes 64 and brown adipocytes 35 were transplanted to the subcutaneous intrascapular region 35,64 or hindlimb muscles 64 of hyperglycemia NOD/SCID mice with non-fasting glucose levels over 250 mg/dL after intraperitoneal injection of 150 mg/kg STZ 35,64. The UCP1-dependent glucose metabolic benefit was not secondary to weight loss and persisted for over 16 weeks 66,75. Apart from additional glucose uptake by the implant itself, implant-induced activation of endogenous BAT and an insulin-independent decrease in glucogenesis in livers also contributed to the decrease in blood glucose levels 34,65,77. Transplantation also increased GLUT4 expression to improve insulin-resistance in adipocytes and skeletal muscles through PPARα activation, as well as improved pancreatic function through PPARα and PPARγ activation 65.

Min et al. derived beige preadipocytes near the newly outgrowth capillaries of human micro-SATs (1g) embedded in Matrigels in the EGM medium. After adipogenic differentiation and 7-day Fsk treatment, beige adipocytes were derived and transplanted (10 million) to the dorsal region of glucose-intolerant NSG mice fed with 2-week HFD. Transplantation decreased fasting glucose levels while increasing glucose tolerance and turnover after 7 weeks. The increase in glucose turnover was partly attributed to the increased glucose uptake of implanted beige adipocytes along with the neuroendocrine effects of the PSCK1-PENK-IL33 signaling pathway (**Figure 4D**) 34. Through NRIP1 disruption by CRIPSR-Cas9, such preadipocytes were further induced to brown-like adipocytes which improved glucose metabolism over 12 weeks after transplantation 66. Similarly, Wang et al. engineered human brown-like adipocytes from SVF-WATs by activating endogenous UCP1 via CRISPR-Cas9. They injected the brown-like adipocytes (15-20 million) into the thoracic-sternum region of obese BALB/c athymic nude mice pretreated with a 2-week HFD with continued HFD treatment after transplantation. Transplantation showed both preventive and long-term therapeutic potentials of T2DM as evidenced by improvements in glucose metabolism at 4 weeks and 12 weeks after transplantation. Mechanistically, apart from implanted brown-like adipocytes, endogenous BAT activated by brown-like adipocyte-produced nitric oxide (NO) also contributed to glucose metabolic benefit (**Table 3**) 77.

Moreover, Wang et al. generated BrAOs from SVF-BATs in thermoreversible PNIPAAm-PEG hydrogels. They injected BrAO containing 1.25 million adipocytes into the kidney capsule of T2DM Rag1 ^-^/^-^ mice established via low-dose STZ (90 mg/kg) injection after 3-month HFD treatment. The fasting glucose level was reduced, accompanied by improved glucose intolerance and insulin-resistance. The safety of transplantation was confirmed as no tumor or non-adipose tissue was found. Moreover, transplantation also inhibited excessive lipid accumulation in endogenous BATs, WATs, and livers and improved the secretion of endogenous adipokines 36, which may also contribute to metabolic benefit as demonstrated in other studies (**Figure 4C**) 65,132. Notably, PRDM16 transduction successfully induced iPSCs of T2DM KK-Ay mice into brown adipocytes, which reduced both serum and urine glucose levels of syngenic hosts after transplantation (**Table 3**) 75. Besides, the medium of beige adipocytes derived from hiPSCs of T2DM patients increased insulin sensitivity and glucose uptake of the primary white adipocytes from the same patients 39. These two studies showed the potential for autologous transplantation of BrAOs and BeAOs to treat T2DM.

### 4.3 Dyslipidemia and non-alcoholic fatty liver disease

Dyslipidemia, often secondary to obesity and T2DM, is characterized by elevated plasma levels of total cholesterol, LDL-cholesterol, or triglycerides, or reduced levels of HDL-cholesterol 133. NAFLD has a global prevalence of 25% and affects over 80% of obese people 134-136. NAFLD includes a disease continuum from steatosis to non-alcoholic steatohepatitis and even cirrhosis and hepatocellular carcinoma 136,137. Mechanistically, hypertrophic adipose tissues usually induce increased lipolysis, inactivation of lipid metabolic enzymes, and lipotoxicity in livers 136,138. Lifestyle and dietary interventions are crucial for managing dyslipidemia and NAFLD. However, long-term maintenance of lifestyle management poses challenges, necessitating the aid of inconvenient combined medications. Drugs like statins and fibrates can further treat dyslipidemia while no therapy has been approved yet for NAFLD 13,136,139. WAOs combined with organ-on-chip technology provide a platform to elucidate the role of adipose tissues in NAFLD progression and screen potential drugs 104,140. Transplantation of brown adipocytes and BrAOs holds the potential to effectively treat dyslipidemia and NAFLD in a mono-modal manner.

Slaughter et al. developed the first human-on-chip model composed of human hepatocytes and human visceral adipocytes utilizing a serum-free, circulating medium to model healthy, diabetic, obese, and pro-inflammatory metabolic conditions. This model investigated the role of adipocyte lipolysis and insulin-resistance in NAFLD, and the crosstalk pattern of adipokines and cytokines exchanged between two organs. It also modeled the disparity of metformin efficacy on NAFLD between preclinical predictions and clinical results, demonstrating its potential for bridging the bench-to-bedside gap in the evaluation of drug efficacy and dose regimens (**Figures 4A-B** and **Tables 1-2**) 104.

Transplantation of brown adipocytes 40,65,75 improved lipid metabolism profile in normal NOD/Shi-*scid IL2rγ*^null^ mice 40 and alleviated dyslipidemia in obese C57BL/6 mice fed with HFD 65 and KK-Ay T2DM mice 75. In detail, the serum lipid pattern was comparable to that of the normal diet group, exhibiting significantly lower levels of total cholesterol, triglyceride 40,65,75, LDL-cholesterol, phospholipid, and non-esterified fatty acids 75, as well as a significantly higher HDL/LDL ratio 65,75 compared to the control group. Furthermore, the olive oral tolerance test confirmed that brown adipocyte transplantation augmented resistance to oral lipid loading 40. Besides, transplantation of brown adipocytes 65,66 demonstrated therapeutic effects on NAFLD in HFD-fed obese C57BL/6 65 and NSG mice 66. From a general perspective, the brown adipocyte group exhibited reduced liver weight and paleness. Histological analysis of the liver revealed decreased accumulation of lipid droplets and hepatocyte ballooning, which was further supported by the quantitative analysis of lipid droplet area, number, and size, as well as determination of liver triglyceride levels. Furthermore, the significantly lower serum levels of glutamate pyruvate transaminase and aspartate transaminase, along with elevated albumin levels indicated improved liver function. Moreover, hepatic inflammation was markedly alleviated with significant downregulation of expressions of pro-inflammatory genes such as MCP-1, TNF-α, IL-1β, TNF-α, and IL-4, as well as upregulation of anti-inflammatory cytokine, IL1-rn, accompanied by suppressed infiltration of F4/80 positive macrophages 65,66. Tsagkaraki et al. generated brown adipocytes from SVF-WATs through NRIP1 disruption by CRISPR-Cas9 and transplanted them into the subscapular region of HFD-treated NSG mice. Livers were smaller and less pale with decreased area, number, and size of lipid droplets, as well as lower gene expressions of CD36 related to fatty acid uptake and pro-inflammatory TNF-α and IL-1β after transplantation than control white groups 66. Lee et al. administrated ADSC-derived brown adipocytes to C57BL/6 mice with steatosis and hepatomegaly through 30 weeks of HFD treatment. Lipid droplet accumulation and hepatocellular ballooning in livers were reversed, along with improved liver function 65. Moreover, the long-term HFD also induced steatohepatitis characterized by inflammation and fibrosis. Transplantation significantly down-regulated expressions of pro-inflammatory TNF-α and IL-4, up-regulated expressions of anti-inflammatory IL1-rn, and repressed F4/80 positive macrophage infiltration for inflammation relief. Moreover, it showed therapeutic effects on liver fibrosis by reducing type I collagen expression and ECM deposition 65. In addition to 2D brown adipocytes, transplantation of BrAOs (1.25 million cells) from SVF-BATs to kidney capsules of T2DM mice also inhibited steatosis progression (**Figure 4C** and **Table 3**) 36.

## 5. Challenges and prospects

### 5.1 Establishment challenges and prospects

Adipose organoids are still in their infancy and have various establishment, bench-to-bedside, and application challenges. There is currently no standardized stepwise strategy with designated intermediate stages like islet organoids to generate adipose organoids from PSCs 35,39,40,60,141. Lineage tracing, RNA-seq, and ATAC-seq can further elucidate development processes in vivo, thereby guiding the stepwise induction in vitro 24,142,143. Besides, beige adipocytes are always mistakenly recognized as brown ones. By characterizing intermediate stages, many 'brown adipocytes' actually undergoing the SplM stage were found to be beige ones 35,40,53,74,144. Proteomics, transcriptomics, and open chromatin analyses are also utilized for identification by comparing with native adipose tissues 35,99,145.

Notably, 3D WAOs have been extensively established and applied to OMDs, whereas most researchers only generated 2D brown or beige adipocytes instead of 3D organoids. Considering the advantages of 3D, 3D fabrication techniques should be further applied to BeAO and BrAO establishments. However, current 3D adipose organoids still failed to fully recapitulate the structure and function of native adipose tissues 146. One of the solutions is to characterize the disparities 35,99 and fill the gaps by improving source choices, induction recipes, and fabrication methods 66,77. In addition to the aforementioned sources, adipogenic fibroblasts such as C3H10T1/2 MSCs 147,148 and 3T3-L1 preadipocytes 83,86,99,112,149, human dermal fibroblasts 75,150,151, and mature adipocytes 43 have also been applied. The progressively richer sources can better meet various needs of adipose organoids for applications. Additionally, the precise control of the application of small molecules at the appropriate stage is crucial. Notably, in the later stage of adipogenic differentiation, rosiglitazone is usually preserved 35,39,57,75, lipids are sometimes supplemented 35,57,91, while IBMX and dexamethasone are sometimes removed 36,38,60,92. Considering the intimate relationship between circadian rhythm and adipose tissue homeostasis 152, particularly BAT 153, core circadian clock activators should be further applied to adipose organoids 154. Meanwhile, vascularization, mechanical modification, and precise size control through microwells or hydrogels also promote maturation by improving adipokine secretion profile and metabolic activity 29,36,73.

Although once considered bland in morphology and function, adipose tissue is now recognized as heterogeneous, dynamic, and plastic. Over 60 subgroups of adipocytes, adipose stem and progenitor cells, preadipocytes, fibroblasts, ECs, and immune cells have been identified 155-157. Therefore, the inclusion of ECs, neurons, and immune cells is crucial for generating mature adipose organoids and investigating intricate cell crosstalk 158. Vascularization and immune cell incorporation in vitro have been well discussed in **Section 3.4**, whereas innervation has only been achieved after transplantation 36. Notably, neuromesodermal progenitors have shown potential in self-organizing innerved mesoderm-derived organoids 159,160. Besides, it is imperative to establish high-fidelity adipose organoids to elucidate the association of depot and subject heterogeneity with different physiologically or pathologically metabolic conditions. Mature adipocyte aggregate cultures, collagen and silk hydrogels, VAT-EC incorporation, and microfluidic systems can maintain depot-specific or subject-specific characteristics without impairing adipogenesis capacity, thereby facilitating the application of adipose organoids in disease modeling and drug screening 38,43,104,105,108,161.

Notably, adipose organoids also exhibited undesired or uncharacterized cell populations. This can be overcome by improving differentiation efficiency through optimized induction recipes, gene engineering techniques 64,66, and cell isolation during differentiation such as CD29 for thermogenic preadipocytes 40,57,81. Many studies lacked quantification of differentiation efficiency. The differentiation efficiency of white adipocytes is usually quantified by lipid droplet staining. For beige and brown adipocytes, UCP1 expression levels should be additionally characterized by immunostaining or flow cytometry 35,64. Notably, flow cytometry may underestimate the efficiency due to the high buoyancy of adipocytes 35. Additionally, batch-to-batch variations in viability, size, differentiation efficiency, and functionality pose challenges in high-throughput research and undermine result credibility 41. Therefore, standardization of establishment protocols is necessary 36.

### 5.2 Bench-to-bedside challenges and prospects

Although hiPSC-derived adipose organoids show great promise in personalized medicine, their tumorigenicity which requires a rigorous evaluation before transplantation blocks further clinical application. Recently, a transient-naive-treatment reprogramming method has shown the potential to overcome this limitation 162. In addition, vascularization, innervation, and minimal immune response are imperative for the long-term survival of functional implants. Besides the aforementioned methods, embedding a catheter at the transplant site directly promoted vascularization in vivo 163. Notably, current transplantations have always been conducted in immunodeficiency or immunocompromised mice which is not applicable to humans. The employment of CRISPR-Cas9 to eliminate human leukocyte antigen expressions and ex vivo interferon-γ stimulation to induce endogenous PD-L1 expressions show promise in solving immune issues 143,164. Furthermore, although scaffold-based strategies show advantages in uniformity for high-throughput research, ethical concerns render their further clinical translation. To overcome this, enzyme digestion for scaffold removal and biosafe hydrogels such as thermoreversible and human-derived hydrogels have been applied 36,107, as well as droplet emulsion microfluidic which generates thousands of size-controlled (250-450 μm in diameter) micro-organospheres 165. For further clinical translation, attention should also be given to serum-free medium to avoid xenogeneic contamination and hydrogels supporting long-term preservation 36,64.

It is noteworthy that transplantation of thermogenic BrAOs and BeAOs shows great potential for the treatment of OMDs. However, it is important to achieve long-term therapeutic effects on metabolic improvement considering the time and effort cost of in vitro construction and in vivo invasive procedures. Although previous in vivo experiments have demonstrated efficacy up to 180 days 36, many researchers have overlooked the importance of emphasizing and exploring long-term efficacy, often limiting observations to only 1-2 weeks after transplantation while disregarding the potential whitening of thermogenic adipose grafts in vivo 35,64. To address this issue, it is crucial to first clarify the nature of brown and beige adipose tissues. BAT, originating from paraxial mesoderm, is a stable thermogenic adipose tissue, whereas beige adipose tissue, emerging from WAT under stimuli, is unstable. Therefore, further elucidation of the in vivo developmental trajectory of BAT is imperative for facilitating the construction of authentic BrAOs in vitro instead of BeAOs which go whitening without browning stimuli in vivo after transplantation. In addition, the current studies that achieved long-term effects are those that transplanted BrAOs rather than brown adipocytes. This may be attributed to the fact that organoids can better facilitate the cell-cell and cell-ECM interactions to support thermogenic phenotype maintenance. In addition, considering sites for transplantation is also noteworthy as regions that naturally distribute thermogenic adipose tissues such as interscapular regions may provide a more favorable microenvironment to avoid whitening.

### 5.3 Application challenges and prospects

WAOs have been mainly applied to disease modeling and drug screening of OMDs. Considering the metabolic healthy adipokine secretion, it should be potential for OMD treatment and did reduce triglyceride levels and improve lipid intolerance 40. Nevertheless, it was also reported to induce glucose intolerance and insulin-resistance 40. Therefore, further studies are required to explore the therapeutic potential of WAOs. BrAOs and BeAOs have been mainly used for transplantation to treat OMDs. However, many studies only listed therapeutic phenomena without elucidation of mechanisms. In addition to the direct effects of implants, their effects on other organs such as endogenous WATs and BATs, livers, skeletal muscles, and central nervous systems possibly through batokines 166 may also contribute to the metabolic benefits 167. Additionally, BeAOs and BrAOs should be further employed in high-throughput screening of novel browning molecules 43,64,69 to enhance ex vivo browning efficiency for transplantation and promote in vivo white-to-beige transition after oral administration 69. Notably, inosine secretion from apoptotic brown adipocytes 168 and damaged mitochondria removal of macrophages 169 were reported to promote thermogenesis in BATs. Therefore, investigations on cell crosstalk in adipose organoids are required. In addition, adipose organoids should be further applied to cirrhosis, hepatocellular carcinoma, and cardiovascular diseases 170, as well as systematic inflammation diseases such as osteoarthritis 113.

## 6. Conclusions

Translational OMD research is significantly hampered by the lack of accurate and reliable in vitro models that can faithfully replicate human physiology and pathology. Meanwhile, transplantation of brown and beige adipose tissues to treat OMD is restricted by their scarcity in vivo. Adipose organoids derived from PSCs, ADSCs, SVF-WATs, SVF-BATs, and micro-WATs provide a promising platform for unraveling underlying mechanisms and developing potential treatments of OMDs. Specificially, WAOs were mainly used for disease modeling and drug screening, while BeAOs and BrAO showed promise in increasing energy expenditure and improving lipid and glucose metabolism upon transplantation. Various scaffold-free and scaffold-based 3D techniques, along with strategies to promote vascularization and immune cell incorporation, have been extensively employed in adipose organoids; however, adipose organoids still fail to fully recapitulating the intricate structure and function of adipose tissues in vivo. In fact, advancements are required to improve the maturity and heterogeneity during establishment, address immune rejection challenges and safety concerns associated with transplantation, as well as expand applications in OMDs for further progress. The continued development of adipose organoids will be a milestone in comprehending and treating OMDs.

## Figures and Tables

**Table 1:** Studies establishing adipose organoids as disease models for obesity-related metabolic diseases

Diseases	Studies	Sources	Methods	Mechanisms	Highlight Results
**Obesity**	Pieters et al. (2022)^25^	hADSCs	Emdebde in fibrin/Geltrex™ hydrogels, and infiltrated into a thin cellulose scaffold	-Exposure to saturated palmitic acids (250 μM, 500 μM, or 750 μM); -Exposure to unsaturated oleic acids (250 μM, 500 μM, or 750 μM)	-Palmitic acid exposure: lipid droplet sizes↑, insulin-sensitivity↑, basal lipolysis↑, secretion of pro-inflammatory cytokines which alter macrophage gene expression↑. -Oleic acid exposure: lipid droplet sizes↑, insulin-sensitivity↑.
	Taylor et al. (2020)^119^	mSVFs (with resident macrophages and monocytes maintained)	Seeded in 96-well ULA plates	-Exposure to saturated stearic acids (0.5mM); -Exposure to unsaturated oleic acids (0.5mM)	-Stearic acid exposure: lipolysis↑, a ratio of neutral to polar lipids↓. -Oleic acid exposure: lipolysis↑, levels of two polyunsaturated triglycerides↑, a ratio of neutral to polar lipids↓.
	Emont et al. (2015)^105^	Visceral and subcutaneous mSVFs	Cultured in collagen hydrogels	Collagen hydrogels support depot-specific characteristic maintenance	-SAT organoids: SAT markers (SHOX2 and TBX15)↑, thermogenic genes (CIDEA, COX7A1, DIO2, PPARGC1A, and PRDM16)↑. -VAT organoids: VAT markers (AGT and WT1)↑, cytokine genes (CCL2, CCL5, IL6, IL10, and TNFA)↑, secretion of cytokines (IL-6 and TNF-α) in response to LPS stimuli↑.
	Aubin et al. (2015)^97^	hADSCs	Superposition of 3 cell sheets	Exposure to TNF-α (10 ng/ml)	-Secretion of MCP-1↑, HGF↑, and NGF↑; NF-κB-dependent genes↑, genes implicated in NF-κB activation↑. -Functionally metabolic genes (GLUT4, FAS, and HSL)↓.
	Proulx et al. (2016)^95^	hADSCs and hMVECs	Superposition of 3 cell sheets	Exposure to TNF-α or IL-1β(10 ng/ml)	-Secretion of pro-inflammatory cytokines (MCP-1, IL-6, and Ang-1)↑. -Microvascular network formation↓.
	Abbott et al. (2018)^78^	Human liquified lipoaspirates from subjects with different BMI	Seeded into silk scaffolds	Exposure to TNF-α (10 ng/ml)	-Glucose uptake↑. -No significant differences in responsiveness of glucose uptake and lipid droplet sizes among WAOs derived from different subjects (BMI = 29, 30, 33, 36, 51 kg/m^2^).
	Harms et al. (2019)^43^	Visceral and subcutaneous adipocytes	Membrane mature adipocyte aggregate cultures	Co-cultured with mice macrophages (RAW264.7) and treated with LPS (10 ng/ml)	-Adipocytes: secretion of IL-6↑ and IL-8↑. -Macrophages: secretion of TNF-α↑ and IL-6↑.
	Taylor et al. (2020)^119^	mSVFs (with resident macrophages, mast cells, and other immune cells miantianed)	Seeded in Nunclon Sphera 96-well ULA U-bottom plates	Exposure to LPS (100 ng/mL)	-Secretion of IL-6↑ and obesity-induced chemokine CCL2↑. -Lipidome changes found by mass spectrometry: diacylglycerol↓, cholesterol↑, no change in triglyceride fraction.
	Rajangam et al. (2016)^121^	hADSCs	Seeded in 96-well ULA plates	Immobilized FGF2 in well surfaces	Fibrosis-related genes (TGF-β1, αSMA, and Col I)↑, synthesis and accumulation of Col I↑, crosslinking of ECM components↑.
**T2DM**	Park et al. (2019)^83^	Mice 3L3-L1 preadipocytes	Suspendended in alginate hydrogel mixture for 3D bioprinting	Co-cultured with mice macrophages (RAW264.7)	-Adipocytes: inuslin-sensitivity markers (GLUT4 and pAKT)↓, a ratio of GLUT4 expressions in plasma membrane to cytosol↓, ISGU↓. -Macrophages: proliferation↑, pro-inflammatory cytokines (TNF-α and IL-1β)↑, F4/80↑, cytochrome C↑. -Levels of adipogenic markers comparable to 3D mono-culture.
	Park et al. (2020)^84^	hADSCs	Suspendended in alginate hydrogel mixture for bioprinting of 3D beads	Loading hADSCs and mice macrophages (RAW264.7) in separate wells of 3D co-culture acrylic plates allowing cell interaction	-Inuslin-sensitivity markers (GLUT4 and pAKT)↓, ISGU↓. -Macrophages proliferating from 1% to 15%. -Adipogenic differentiation capacity compared to 3D mono-culture.
	Choi et al. (2010)^116^	hADSCs and HUEVCs	Co-cultured in 3D silk scaffolds	High concentration of insulin (10 mM) exposure	-Triglyceirde accumulation↑. -Glycerol release↓.
	Acosta et al. (2022)^38^	MVFs from T2DM and lean rats	Embeded in fibrin hydrogels	Fibrin hydrogels support subject-specific characteristic maintenance	-Db-MVF-derived WAOs: vessel structure size↓, vessel connections↓, ISGU↑, comparative levels of isoproterenol-stimulated lipolysis and OCR. -Db-MVF-derived BrAOs: vessel structure size↓, vessel connections↓, UCP1 expression↑, maximum respiration↑, spare capacity↑, comparative levels of ISGU and isoproterenol-stimulated lipolysis.
**NAFLD**	Slaughter et al. (2021)^104^	Primary human cardiac preadipocytes and primary human hepatocytes	Incorporated into two-chamber housing system	-Healthy plasma condition: post-prandial glucose (5 mM) and insulin (1 nM); -Diabetic plasma condition: post-prandial glucose (25 mM) and insulin (69 nM); -Pro-inflammatory plasma condition: TNF-α (10 µM); -Obese plasma condition: 45 μM BSA-conjugated palmitate and 65 μM oleate	-Steatosis↑ in diabetic medium with or without lipids and obese medium with and without TNF-α. -CYP3A4 activity↓ and adiponectin secretion↓ in proinflammatory medium alone and plus obese medium. -Leptin secretion↑ (slightly) and adiponectin to leptin ratio↓ in diabetic plus proinflammatory medium with and without lipids. -No significant changes in steatosis in pro-inflammatory medium and adiponectin secretion in diabetic and obese medium.

Alginate hydrogel mixture: containing 20 mg/mL alginate, 0.5 mg/mL gelatin, and 0.5 mg/mL Col I; Ang-1: angiopoietin-1; αSMA: α-smooth muscle actin; BMI: body mass index; BSA: bovine serum albumin; CIDEA: cell death-inducing DNA fragmentation factor alpha-like effector A; Col I: type I collagne; COX7A1:cytochrome c oxidase subunit 7A1; Db-MVF: microvascular fragments isolated from subcutaneous adipose tissues from diabetic rats; DIO2: iodothyronine deiodinase 2; ECM: extra cellular matrix; FAS: fatty acid synthase; GLUT4: glucose transporter 4; hADSC: human adipose-derived stem cell; HGF: hepatocyte growth factor; hMVEC: human microvascular endothelial cell; HSL: hormone-sensitive lipase; HUEVC: human umbilical vein endothelial cells; IL: interleukin; ISGU: insulin-stimulated glucose uptake; LPS: lipopolysaccharide; MCP-1/CCL2: monocyte chemoattractant protein-1/C-C Motif Chemokine Ligand 2; mSVF: mice stromal vascular fraction; MVF: microvascular fragments; NF-κB: nuclear factor kappa B; NGF: nerve growth factor; WAO: white adipose organoid; OCR: oxygen consumption rate; pAKT: AKT phosphorylation; PPARGC1A: peroxisome proliferator-activated receptor γ coactivator-1α; PRDM16: PR domain-containing 16; RANTES (CCL5): regulated-on-activation-normal-T-Cell-expressed-and secreted; SAT: subcutaneous adipose tissue; TGF-β: tumor growth factor beta; TNF-α: tumor necrosis factor-alpha; T2DM: type 2 diabetic mellitus; UCP1: uncoupled protein 1; ULA: ultra-low attachment; VAT: visceral adipose tissue; 3D: three-dimensional.

**Table 2:** Studies establishing adipose organoids as drug screening platforms for obesity-related metabolic diseases

Diseases	Studies	Sources	Methods	Efficiency validation	Screening drugs	Highlight results	In vivo validation
**Obesity**	Choi et al. (2022)^122^	mice 3T3-L1 preadipocytes and macrophages (RAW264.7)	-Suspended in alginate hydrogel mixture for bioprinting of 3D beads; -Tested in 96-well platforms	-Rosi: lipid accumulation↑; -GW9662: lipid accumulation↓	178 compounds consisting of AMPK activators, PPARγ agonists, and PPARγ antagonists	-1^st^ hit: 7 compounds: adipogenesis↓ in 3D instead of 2D. -2^nd^ hit: 3 compounds: repetitive anti-adipogenic effects in 3D, adiponectin expression↑; Compound #71: pro-inflammatory cytokines (TNF-α, IL-6, and IL-1β)↓.	None
	Abbott et al. (2018)^78^	Human liquified lipoaspirates from ten subjects with different BMI	-Seeded into silk scaffolds; -Tested in 24-well platforms	None	AICAR (500 μM): one of exercise mimetics	-Lipolysis↓ in only 3 of the 10 patient samples. -Responsiveness was not correlated with BMI (varied from 22, 23, 23, 26, 30, 32, 32, 33, 33, 51 kg/m^2^).	None
**T2DM**	Park et al. (2019)^83^	mice 3T3-L1 preadipocytes and macrophages (RAW264.7)	-Suspended in alginate hydrogel mixture for 3D bioprinting	Rosi: glucose uptake↑, GLUT4 expression↑, G6PD enzyme activity↓	5 PPARγ antagonists: GW9662, BADGE, SR202, FH535, and T0070907	-1^st^ hit: All 5 antagonists: lipogenesis-related genes and proteins (FABP4, ADIPOQ, PLIN, and PPARγ2)↓. -2^nd^ hit: GW9662 (10 μM): glucose uptake↑, GLUT4 expression↑, G6PD enzyme activity↓.	C57BL/6*^ob/ob^* mice orally administrated GW9662 (300 mg/kg): -Body and fat weights↓. -OGTT↑, ITT↑, HOMA-IR index↓.
	Park et al. (2020)^84^	hADSCs and macrophages (RAW264.7)	-Suspended in alginate hydrogel mixture for bioprinting of 3D beads; -Loaded in separate wells of 3D co-culture acrylic plates allowing cell interaction	Metformin and Rosi: glucose uptake↑, adiponectin secretion↑	11β-HSD1 inhibitors: KR-1 (mouse-potent enantiomer), KR-2 (human-potent enantiomer) KR-3 (racemic compound)	-1^st^ hit: KR-1 and KR-3: mouse 11β-HSD1↓; KR-2: human 11β-HSD1↓. -2^nd^ hit: KR-1 and KR-3: glucose uptake↑, GLUT4 expression↑ in mice-derived WAOs; KR-2 and KR-3: glucose uptake↑, GLUT4 expression↑ in human-derived WAOs.	None
**NAFLD**	Slaughter et al. (2021)^104^	Primary human cardiac preadipocytes and primary human hepatocytes	Incorporated into two-chamber housing system	None	Metformin	Metformin (1mM) for a shortened 7-day treatment: -2D: Cell death. -3D: Steatosis↓ in diabetic, obese, diabetic plus obese, diabetic plus obese and proinflammatory medium.	Failure in clinical translation: The result dose is above physiological range in clinical application (1-50 μM).

ADIPOQ: adiponectin; AICAR: 5‐aminoimidazile‐4‐caboxamide‐1‐β‐D‐ribofuranoside; AMPK: adenosine monophosphate-activated protein kinase; BADGE: Bisphenol A diglycidyl ether; BMI: body mass index; FABP4: fatty acid binding protein 4; GLUT4: glucose transporter 4; GW9662: PPARγ antagonist; G6PD: glucose-6-phosphate dehydrogenase; hADSC: human adipose-derived stem cells; HOMA-IR: Homeostatic Model Assessment for Insulin Resistance; IL: interleukin; ITT: insulin tolerance test; KR: compound synthesized at the Korean Research Institute of Chemical Technology; OGTT: oral glucose tolerance test; PLIN: perilipin; PPARγ: peroxisome proliferator-activated receptor gamma; Rosi: rosiglitazone; TNF-α: tumor necrosis factor-alpha; WAO: white adipose organoid; 3D: three-dimensional; 11β-HSD1: 11β-hydroxysteroid dehydrogenase type 1.

**Table 3:** Studies establishing adipose organoids for transplantation to treat obesity-related metabolic diseases

Diseases	Studies	Sources	Methods	Amount	Host	Region	Highlight Results	Mechanisms
**Obesity**	Lee et al. (2017) 65	hADSCs	Supplemented with transferrin (10 μg/ml), T3 (0.2 nM), and Rosi (100nM) to promote browning	1 million brown adipocytes/kg per 2 weeks for 10 weeks	C57BL/6J mice fed with 30-week HFD	Intraperitoneal	-Body weight↓. -TNF-α↓, IL-1↓, adiponectin-to-leptin ratio↑.	Unexplored
	Blumenfeld et al. (2018)^73^	Micro-WATs from HFD-fed C57BL/6 mice	Supplemented with T3 (250 nM), Rosi (1 μM), CL316243 (1 μM), and VEGF (25 ng/ml) for 3 weeks to promote ex vivo browning	0.2 ml BrAOs	HFD-fed C57BL/6 mice	Right rear hindlimb	-Body weight↓, fat mass↓ (significant at 15 weeks). -No significant differences in VO2 and EE at 1 week.	Unexplored
	Singh et al. (2020)^64^	hADSCs	Supplemented with transferrin (10 μg/ml), T3 (1 nM), Rosi (2 μM), Y-27632 (10 μM), and BMP7 (100ng/ml) to promote browning	2 million beige adipocytes	STZ-induced hyperglycemia NOD/SCID mice	Hindlimb muscle on both sides	-Body weight gain↓; -VO2↑, EE↑, core T↑.	Non-shivering thermogenesis to increase energy expenditure
	Wang et al. (2023)^36^	Immortalized hSVF-BATs	-PNIPAAm-PEG hydrogels promote uniformity of 3D BrAOs (100μm in diameter); -Supplemented with EBM-2, SB-431542 (5 μM), T3 (0.2 nM), and Rosi (1 μM) to enhance brown differentiation efficiency	1.25 million brown adipocytes in BrAO	Rag1^-^/^-^ mice fed with HFD for 3 months followed with low-dose STZ (90 mg/kg) injection	Kidney capsule	-Body weight gain↓, fat content↓. -UCP1 expression miantained for at least 5 months.	Unexplored
**T2DM**	Min et al. (2016)^34^	Human micro-SATs (1g)	-Embedded in Matrigels in EGM2-MV for angiogenesis; -Supplemented with EGM2-MV for 10-day differentiation and treated with Fsk (50 μM) for 7-day brown induction	10 million beige adipocytes	Glucose intolerant NSG mice through 2-week HFD treatment	Dorsal	-Fasting glucose level↓. -Glucose tolerance↑, glucose turnover↑ at 7 weeks.	Glucose turnover↑: -Extra glucose uptake of implants; -Neuroendocrine effects of the PSCK1-PENK-IL33 signaling pathway
	Lee et al. (2017) 65	hADSCs	Supplemented with transferrin (10 μg/ml), T3 (0.2 nM), and Rosi (100nM) to promote browning	1 million brown adipocytes/kg per 2 weeks for 10 weeks	C57BL/6J mice fed with 30-week HFD	Intraperitoneal	-Fasting glucose level↓, insulin-sensitivity↑, glucose tolerance↑. -Glucose uptake in endogenous adipocytes and skeletal muscles↑. -Glucogenesis in livers↓. -Function and insulin-sensitivity of islets↑.	-GLUT4↑, PPARα↑ in endogenous adipocytes and skeletal muscles; -GLUT2↓, G6PC↓ in livers; -PPARα↑, PPARγ↑ in islets
	Tsagkaraki et al. (2021)^66^	-mSVF-WATs; -human preadipocytes^34^	Supplemented with Rosi (1 μM) and transfected with Cas9/ sgRNA RNPs to disrupt NRIP1 to promote browning	Brown adipocytes from a 1 × 150 mm fully confluent plate	NSG mice fed with HFD after transplantation	Subscapular	-Fasting glucose level↓ at 12 weeks. -Glucose intolerance↓ last over 15 weeks. -Maintenance of UCP1 expression for 16 weeks.	Unexplored
	Wang etal. (2020)^77^	hSVF-WATs	Supplemented with T3 (2 nM) and activate endogenous UCP1 by CRISPR-Cas9 to promote browning	15-20 million HUMBLE cells	Obese BALB/c athymic nude mice pretreated with 2-week HFD and continued HFD after transplantation	Thoracicsternum	-Preventive potential: glucose intolerance↓, insulin-resistance↓, insulin concentrations↓ at 4 weeks. -Long-term therapeutic potential: glucose intolerance↓, maintenance of UCP1 at 12 weeks.	NO produced by HUMBLE cells activated endogenous BATs: BAT-selective markers↑, glucose or fatty acid metabolism markers↑, glucose uptake↑
	Tsunao et al. (2015)^75^	Mice fibroblasts	Transduced with PRDM16, C/EBPB, and L-MYC and supplemented with T3 (1 nM) and Rosi (1 μM) to promote the direct conversion into dBAs	dBAs from 2 confluent 100-mm dishes	C57BL/6 mice fed with HFD	Subcutaneous flank	Glucose tolerance↑, insulin-sensitivity↑ as early as 2 weeks after transplantation before weight loss.	Not secondary results of suppression of weight gain
	Zhang et al. (2020)^35^	hiPSCs	-Supplemented with NOGGIN (50 ng/ml), Fsk (5 μM), WNT3a (25 ng/ml), and BIO (2 μM) for paraxial mesoderm specification; -Supplemented with Rosi (2 μM), BMP7 (100 ng/ml), T3 (1 nM), Y-27632 (10 μM), and SB-431542 (10 μM) to promote browning	5 million brown adipocytes	Hyperglycemia NOD/SCID mice through 150 mg/kg STZ injection	Subcutaneous intrascapular	Non-fasting glucose level↓ over 7 days after transplantation, glucose intolerance↓.	GLUT1↑, glucose uptake↑, glycosis↑ of implants
	Singh et al. (2020)^64^	hADSCs	Supplemented with transferrin (10 μg/ml), T3 (1 nM), Rosi (2 μM), Y-27632 (10 μM) and BMP7 (100ng/ml) to promote browning	2 million beige adipocytes	Hyperglycemia NOD/SCID mice through 150 mg/kg STZ injection	-Subcutaneous intrascapular; -Hindlimb muscle on both sides	Non-fasting glucose level↓ over 5 days after transplantation, glucose intolerance↓.	Glucose uptake↑ of implants
	Wang et al. (2023)^36^	Immortalized hSVF-BATs	-PNIPAAm-PEG hydrogels promote uniformity of 3D BrAOs (100μm in diameter); -Supplemented with EBM-2, SB-431542 (5 μM), T3 (0.2 nM), and Rosi (1 μM) to enhance brown differentiation efficiency	1.25 million brown adipocytes in BrAO	Rag1^-^/^-^ mice fed with HFD for 3 months followed with low-dose STZ (90 mg/kg) injection	Kidney capsule	Fasting glucose level↓, glucose intolerance↓, insulin-resistance↓.	-Protect BATs, WATs, and livers from lipotoxicity; -Extra metabolic healthy adipokines (adiponectin, chemerin, and TGF-β) secreted by implants; -Endogenous adipokine secretion↑
**Dyslipidemia and NAFLD**	Tsunao et al. (2015)^75^	-iPSCs derived from fibroblasts of T2DM KK-Ay mice; -fibroblasts from mice	iBAs: -Transduced with PRDM16; dBAs: -Transduced with PRDM16, C/EBPB, and L-MYC; -Supplemented with T3 (1 nM) and Rosi (1 μM) to promote the direct conversion into dBAs	iBAs or dBAs from 2 confluent 100-mm dishes	-iBAs: KK-Ay T2DM mice; dBAs: KK-Ay T2DM mice, HFD-fed C57BL/6 mice	Subcutaneous flank	TC↓, LDL cholesterol↓, triglyceirde↓, phospholipid↓, NEFA↓, HDL/TC ratio↑.	Unexplored
	Lee et al. (2017) 65	hADSCs	Supplemented with transferrin (10 μg/ml), T3 (0.2 nM) and Rosi (100nM) to promote browning	1 million brown adipocytes/kg per 2 weeks for 10 weeks	C57BL/6J mice fed with 30-week HFD	Intraperitoneal	-Dyslipidemia: TC↓, TG↓, HDL/TC ratio↑. -NAFLD (Steatosis): lipid droplet accumulation↓, hepatocellular ballooning↓, liver function↑ (GPT↓, AST↓, Alb↑). -NAFLD (NASH): inflammation↓ and fibrosis↓ in livers.	-Inflammation relief: pro-inflammatory cytokines (TNF-α and IL-4)↓, anti-inflammatory cytokines (IL1-rn) ↑, F4/80^+^ macrophage infiltration↓; -Fibrosis relief: Col I↓, ECM deposition↓
	Tsagkaraki et al. (2021)^66^	mSVF-WATs; huma preadipocytes^34^	Supplemented with Rosi (1 μM) and transfected with Cas9/ sgRNA RNPs to disrupt NRIP1 to promote browning	Brown adipocytes from a 1 × 150 mm fully confluent plate	NSG mice fed with HFD after transplantation	Subscapular	Smaller and less pale liver with lipid droplet area↓, number↓, size↓, TG↓.	Genes related to fatty acid uptake (CD36)↓ and inflammation (MCP-1, TNF-α, and IL-1β)↓ in livers

Alb: albumin; AST: aspartate transaminase; BAT: brown adipose tissue; BMP7: bone morphogenetic protein 7; BrAO: brown adipose organoid; CL316243: mice β3 adrenoreceptor agonist; Col I: type I collagen; dBA: fibroblasts directly converted into BAs by gene transduction; EBM-2: endothelial base medium 2; ECM: extra cellular matrix; EE: energy expenditure; EGM2-MV: endothelial cell growth medium-microvascular; Fsk: forskolin; GLUT: glucose transporter; GPT: glutamate pyruvate transaminase; G6PC: glucose-6-phosphatase; hADSC: human adipose-derived stem cell; HDL: high-density lipoprotein; HFD: high-fat diet; hSVF-BAT: human stromal vascular fragment from brown adipose tissue; HUMBLE: human brown-like; iBA: iPSC-derived embryoid bodies induced BA phenotypes; IL: interleukin; iPSC: induced pluripotent stem cell; LDL: low-density lipoprotein; MCP-1: monocyte chemoattractant protein-1; mSVF-WAT: mice stromal vascular fragement of white adiose tissue; NAFLD: non-alchoholic fatty liver disease; NASH: steatohepatitis; NEFA: non-essential fatty acid; NO: nitric oxide; NOD/SCID: non-obese diabetic/severe combined immunodeficiency; NSG: NOD-*scid IL2rγ^null^*; PENK: proenkephalin; PNIPAAm-PEG: poly(N-isopropylacrylamide)-poly(ethylene-glycol); PPARα: peroxisome proliferator-activated receptor alpha; PPARγ: peroxisome proliferator-activated receptor gamma; PRDM16: PR domain-containing 16; PSCK1: proprotein-convertase subtilisin/kexin type-1; Rag1 ^-^/^-^: Rag1 knock-out; RNP: ribonucleoprotein; Rosi: rosiglitazone; SAT: subcutaneous adipose tissue; sgRNA: single-guide RNA; STZ: streptozotocin; T: temperature; TC: total cholesterol; TG: triglyceride; TGF-β: tumor growth factor beta; TNF-α: tumor necrosis factor-alpha; T2DM: type 2 diabetes mellitus; T3: 3,3',5-Triiodo-L-thyronine sodium salt; UCP1: uncoupled protein 1; VEGF: vascular endothelial growth factor; VO2: maximum volume of oxygen; WAT: white adipose tissue; 3D: three-dimensional.

## Abbreviations

3D: three-dimensional
BAT: brown adipose tissue
BeAO: beige adipose organoid
BrAO: brown adipose organoid
C/EBPα: CCAAT/enhancer binding protein α
dECM: decellularized extracellular matrix
EB: embryonic body
EC: endothelial cell
ECM: extracellular matrix
ELP: elastin-like polypeptide
GelMA/HAMA: methacrylate gelatin and hyaluronic acid
HFD: high-fat diet
IBMX: 3-Isobutyl-1-methylxanthine
ISGU: insulin-stimulated glucose uptake
LPS: lipopolysaccharide
MSC: mesenchymal stem cell
MVF: microvascular fragment
NAFLD: non-alcoholic fatty liver disease
NOD/SCID: non-obese diabetic/severe combined immunodeficiency
NSG: NOD-*scid IL2rγ^null^*
OCR: oxygen consumption rate
OMD: obesity-related metabolic disease
PEI: polyethyleneimine
PLGA: poly(lactic-co-glycolic acid)
PNIPAAm-PEG: poly(N-isopropylacrylamide)-poly(ethylene-glycol)
PPARγ: peroxisome proliferator-activated receptor γ
SAT: subcutaneous adipose tissue
SplM: splanchnic mesoderm
SVF: stromal vascular fragment
STZ: streptozotocin
SVF-WAT: stromal vascular fragment from white adipose tissue
T2DM: type 2 diabetes mellitus
T3: triiodothyronine
UCP1: uncoupling protein 1
VAT: visceral adipose tissue
WAO: white adipose organoid
